# Characteristics of the oral microbiome in youth exposed to caregiving adversity

**DOI:** 10.1016/j.bbih.2024.100850

**Published:** 2024-08-28

**Authors:** Naomi N. Gancz, Francesca R. Querdasi, Kristen A. Chu, Emily Towner, Eason Taylor, Bridget L. Callaghan

**Affiliations:** University of California, Los Angeles, 502 Portola Plaza, Los Angeles, CA, 90095, United States

**Keywords:** Microbiome, Oral microbiome, Adversity, Adoption, Stress, Cortisol

## Abstract

Caregiving adversity (CA) exposure is robustly linked to increased risk for poor oral, physical, and mental health outcomes. Increasingly, the gut microbiome has garnered interest as a contributor to risk for and resilience to such health outcomes in CA-exposed individuals. Though often overlooked, the *oral* microbiome of CA-exposed individuals may be just as important a contributor to health outcomes as the gut microbiome. Indeed, outside the context of CA, the oral microbiome is well-documented as a regulator of both oral and systemic health, and preliminary data suggest its association with mental health. However, research examining the association between CA and the oral microbiome is extremely sparse, especially in childhood, when the community composition of such organisms is still stabilizing. To address that sparsity, in the current study, we examined composition and differential abundance metrics of the oral microbiome in 152 youth aged 6–16 years, who had either been exposed to significant caregiving adversity (significant separation from or maltreatment by a caregiver; N = 66, CA) or who had always remained with their biological/birth families (N = 86, Comparison). We identified a significant negative association between hair cortisol and oral microbiome richness in the Comparison group that was significantly blunted in the CA group. Additionally, youth in the CA group had altered oral microbiome composition and elevated abundance of potentially pathogenic bacteria relative to youth in the Comparison group. Questionnaire measures of fatigue, somatic complaints, and internalizing symptoms had limited associations with oral microbiome features that were altered in CA. Although we found differences in the oral microbiomes of CA-exposed youth, further research is required to elucidate the implications of those differences for health and well-being.

## Highlights

• Adversity is associated with altered oral microbiome composition in youth.
• Adversity blunts the negative association between cortisol and microbiome richness.
• Limited associations between the microbiome and internalizing symptoms are seen.

## 1 Introduction

Early adversity (EA), defined as exposure to chronic or severe stressors prior to adulthood, is linked to a variety of health risks. These include, but are not limited to, poor oral health (Boyce et al., 2010; Sarvas et al., 2021; Bright et al., 2015), immune dysregulation (Danese and McEwen, 2012; Dutcher et al., 2020; Slopen et al., 2013a), cardiovascular disease (Doom et al., 2017), and psychopathology (Conway et al., 2018). Caregiving adversity (CA), a severe subtype of early adversity in which an individual is maltreated by or extensively separated from their caregiver (e.g., due to orphanage or foster care), is an especially concerning risk factor that exerts pronounced effects on stress and immune physiology (reviewed by Kuhlman et al. (2017)), as well as on oral health (Sarvas et al., 2021). Though many compelling theoretical models have been proposed to explain the wide-reaching effects of caregiving adversity on health, very little mechanistic information is known about those associations. The elucidation of such mechanisms is necessary for the development of interventions to reduce risk for the spectrum of harmful outcomes associated with CA exposure.

Although CA is a primarily psychosocial exposure, it affects multiple tissues and organ systems across the body; therefore, the most likely candidate mechanisms underlying the effects of EA on health are those that also exert wide-reaching effects on the body. The microbiome (communities of microorganisms inhabiting niches in the human body) is compelling in this regard because it exerts effects not only in within tissues directly colonized by microbes (Hajishengallis, 2015), but also across the periphery –the cardiovascular system (Gaetti-Jardim et al., 2009; Kebschull et al., 2010), placenta (Gomez-Arango et al., 2017a), and bone tissue (Mikuls et al., 2014; Kinloch et al., 2011)– and even the central nervous system (Riviere et al., 2002; Xue et al., 2020). Indeed, emerging evidence suggests that microbiome dysregulation is a likely mechanism by which early adversity exposure affects individual outcomes (Callaghan et al., 2016, 2020a; Cowan et al., 2019; Vogel et al., 2020; Querdasi et al., 2023; Hantsoo et al., 2020; Reid et al., 2021). Further characterization of microbiome dysregulation following early adversity could lead to the development of treatments, such as prebiotic supplementation, that promote well-being in those exposed to CA.

Links between host psychosocial factors and the *gut* microbiome have recently become popularized among behavioral and neural scientists. However, this focus on the gut microbiome may have led to a neglect of other important microbial habitats which are also related to health outcomes, such as the *oral* cavity. Microorganisms which inhabit the oral cavity comprise the second largest community of microbes in the human body after the gut. They are seated at the primary gateway for toxin, nutrient, and pathogen entry to the human body, making this an important site of influence for health outcomes. Indeed, extensive evidence indicates that the oral microbiome is associated with oral health outcomes, likely causally, as well as with systemic health outcomes. Within the oral cavity, ecological dysregulation of the microbiome directly causes cavities (Struzycka, 2014) and periodontitis (Hajishengallis, 2015). Beyond the oral cavity, dysregulation of the oral microbiome is positively associated with the incidence and severity of cardiovascular disease (Carrion et al., 2012; Kapil et al., 2013), arthritis (Mikuls et al., 2014; Kinloch et al., 2011), Alzheimer's disease (Aragón et al., 2018; Poole et al., 2013), and diabetes (Cho et al., 2021; Taylor et al., 2013; Xiao et al., 2017). Emerging evidence suggests that the abundance of certain taxa in the oral microbiome may be associated with internalizing symptoms, and that these associations are moderated by levels of salivary cortisol, a stress biomarker, and C-reactive protein (CRP), an inflammation biomarker (Simpson et al., 2020; Wingfield et al., 2021). Therefore, elucidation of factors that may induce oral microbiome dysregulation is critical to the promotion of physiological and behavioral health.

Unlike the gut microbiome, the oral microbiome appears to be particularly resistant to antibiotic insult (Wu et al., 2020; Zaura et al., 2015) in adults, suggesting greater stability in this community over time. The oral microbiome also regulates immunity (Hajishengallis, 2015) through pathways distinct from the gut microbiome. As the oral microbiome may be especially amenable to minimally invasive treatment, such as toothpaste additives (Kong et al., 2021), its inclusion in preventative healthcare strategies is likely more feasible than some gut microbiome-based treatments. For these reasons, understanding how environmental variables influence the oral microbiome will be essential for creating new and effective treatments which target the manifold health outcomes associated with this microbial community.

Caregiving adversity is especially concerning as a risk factor for oral microbiome dysregulation. Poor oral hygiene and subsequent disease are highly prevalent in CA-exposed children, so much so that clinicians often use oral health as signs of possible maltreatment (Håkstad et al., 2024). Indeed, adolescents with a history of foster care are more likely to report oral health problems and barriers to receiving dental care (Sarvas et al., 2021; Ferrara et al., 2013), which are robustly linked to oral microbiome dysregulation. CA is also associated with other health behaviors that could influence the oral microbiome, such as diet, in adults (Marquez et al., 2021). However, as caregivers play a larger role in regulating the diet of children, diet may play less of a moderating role for CA impacts on the oral microbiome earlier in development, particularly if children's CA has ended, e.g., they are in a stable caregiving arrangement. In terms of physiological mechanisms, CA is linked to various outcomes likely to affect the oral microbiome, including inflammation (Kuhlman et al., 2020a) and alterations to saliva composition (Kuras et al., 2017).

Not surprisingly, given the behavioral and physiological associates of CA just discussed, young adults who retrospectively reported a history of exposure to early caregiving adversity exhibit differences in oral microbiome composition compared to those with low exposures (Charalambous et al., 2021). Critically, this association is present even when the CA occurred decades prior to the oral microbiome sampling (Charalambous et al., 2021), suggesting that CA exerts a lasting impact on the oral microbiome. Interestingly, CA exposed individuals are also at heightened risk for socioemotional problems, including internalizing symptoms like anxiety and depression (Gardner et al., 2019), and broad physical health problems (e.g., cardiovascular disease (Ho et al., 2020), gastrointestinal distress (Callaghan et al., 2020b)). How early in development changes to the oral microbiome arise after CA exposure and whether they are directly linked to health outcomes in the same individuals remain open questions. As the age of onset for many psychiatric disorders is early adolescence (Kessler et al., 2005), and because the oral microbiome is developing and stabilizing across early childhood and into adolescence (Burcham et al., 2020; Willis et al., 2022), determining the developmental trajectory of microbiome changes after CA is a public health priority with clear basic science importance.

In addition to its direct effects, caregiving adversity may also affect the responsiveness of the oral microbiota to host cortisol levels. Such an effect has been observed in host tissue, wherein chronic CA-induced dysregulation of cortisol levels can cause brain tissue and immune cells to exhibit insensitivity to the regulatory effects of cortisol (McGowan et al., 2009; Tyrka et al., 2012). We posit that, similarly to host tissue, the oral microbiota may become less sensitive to the effects of host-derived cortisol after CA exposure. Cortisol has been shown to exert dysregulatory effects on the oral microbiome in vitro and in typically developing children (Boyce et al., 2010; Duran-Pinedo et al., 2018; Tikhonova et al., 2018), but the association between oral microbiome characteristics and tonic cortisol levels in CA-exposed youth has yet to be studied. Characterizing the effect of CA on the association between tonic cortisol levels (which can be measured in accumulated hair concentrations, for example) and microbiome characteristics will reveal more about the putative mechanisms underlying health in CA-exposed youth.

The current study tests whether CA is associated with compositional characteristics of the oral microbiome in children and adolescents, and whether CA moderates the association between tonic cortisol levels and oral microbiome composition. To probe the potential clinical utility of our findings, we also examined associations between the oral microbiome and health outcomes in youth. Specifically, we examined subclinical and transdiagnostic mental and physical health symptoms which would be more likely to present in a developmental context: fatigue, somatic complaints, and internalizing symptoms. Notably, each of these adverse health outcomes have been shown to be elevated in individuals exposed to early adversity, including specifically CA exposed individuals, (Callaghan et al., 2020b; Orendain et al., 2023; Heim et al., 2006; Crawley et al., 2012) and have been linked to the oral microbiome in prior studies in youth (Simpson et al., 2020) and adults (Wang et al., 2018; Gonzalez et al., 2016; Fourie et al., 2016).

## 2 Materials & methods

### 2.1 Methods

#### 2.1.1 Participants

Participants were enrolled in a larger study, the *Mind, Brain, Body* study, at the University of California, Los Angeles (UCLA), between November 2019–March 2022. Participants who took part in the research before the COVID-19 related research ramp down at UCLA (March 2020) came into the research lab for their first visit, and then completed additional biological sample collection, questionnaires, and a behavioral task at home 2 weeks later. Participants who took part in the research after the COVID-19 related research ramp down at UCLA completed two virtual visits online and mailed their biological samples back to the lab. The parent study included a large number of questionnaires, several biological samples (saliva, stool, blood, hair), a parent-child interaction task that was filmed, and a computerized behavioral task. The current study uses data from the questionnaires, saliva sample, and hair sample.

Informed consent was obtained from participants' caregivers, and assent was obtained from the child and adolescent participants. There were two participant groups: the caregiving adversity (CA) group and the Comparison group. To recruit a sufficiently large CA group, we focused our recruitment efforts on foster youth who had been adopted or placed in guardianship care, as the reasons for foster care placements typically involve some form of caregiver maltreatment (which constitute a CA exposure (U.S. Children's Bureau, 2022)). To be included in the CA group, participants must have experienced some form of caregiver maltreatment or extensive parent separation (as described in the *Caregiving Adversity* section below), and have been between the ages of 6–16 years old at the time of study participation. Youth in the Comparison group must not have endorsed any caregiving related adversities, and must not have had a diagnosed mental illness or learning disability aside from attention deficit disorder (ADD), which was not an exclusion criterion if it was judged by the parent to be unlikely to interfere with study participation, or if the child was taking their prescribed ADD medication at the time of study participation. Youth in the Comparison group must have been either 6–16 years old at the time of study (the original criterion was either 6–9 or 13–16 years old but the age range was expanded due to recruitment challenges). Participants were not enrolled in either the CA or Comparison group if the participant's caregiver reported that the participant had uncorrected vision problems that would interfere with their ability to read or understand study materials/tasks, had used antibiotics in the past 4 months (in this case they were placed on a waitlist and were allowed to enroll in the study when they no longer met the antibiotic exclusion criteria), or the participant smoked marijuana more than once a week, had more than 10 alcoholic drinks per week, or used any other illicit substances, based on parent report.

All individuals from the Mind, Brain, Body study who completed a saliva sample were included in the current analyses, resulting in a final sample size of 66 in the CA group and 86 in the Comparison group. Family-level clustering among siblings was present, but uncommon, in our sample (with an average sibling cluster size of 1.36, see Table 1 for group averages of siblings included in the study). To estimate the effect of this clustering on microbiome diversity and composition, we calculated the design effect of family-level clustering on alpha diversity. The effect was less than 1.15 for Faith's diversity and observed feature counts, and less than 1.08 for Pielou's evenness and Shannon's diversity. Given that these effects are small (representing a sampling variability inflation of about 8–15%), that we are not interested in family-level effects, and that we don't expect effects to vary across families, we consider this negligible (Lai and Kwok, 2015), and therefore, our analyses do not adjust for clustering within families.

**Table 1:** Sample demographics & covariates.

	Race/Ethnicity Counts (and Percentages)		*Total*	
	*Comparison*	*Caregiving Adversity*		
Asian	23 (26.74%)	11 (16.67%)	34 (22.37%)	p = .441
Black/African American	3 (3.49%)	1 (1.52%)	4 (2.63%)	
Multiracial	16 (18.60%)	7 (10.61%)	23 (15.13%)	
Native American	1 (1.16%)	1 (1.52%)	2 (1.32%)	
White (Hispanic)	16 (18.60%)	10 (15.15%)	26 (17.11%)	
White (Not Hispanic)	19 (22.09%)	21 (31.82%)	40 (26.32%)	
No Response	8 (9.30%)	15 (22.73%)	23 (15.13%)	

	Mean Age in Years (and Standard Deviation)		*Total*	
	*Comparison*	*Caregiving Adversity*		
Mean Age in Years (SD)	11.36 (3.64)	11.15 (3.08)	11.27 (3.40)	p = .691

	Sex Counts (and Percentages)		*Total*	
	*Comparison*	*Caregiving Adversity*		
Female	40 (46.51%)	35 (53.03%)	75 (49.34%)	p = .527
Male	46 (53.49%)	31 (46.97%)	77 (50.66%)	

	Feeding Style in Infancy^1^ Counts (and Percentages)		*Total*	
	*Comparison*	*Caregiving Adversity*		
Breastmilk	55 (63.95%)	6 (9.09%)	61 (40.13%)	p < .000*
Breastmilk & Formula	23 (26.74%)	3 (4.55%)	26 (17.11%)	
Formula	8 (9.30%)	33 (50.00%)	41 (26.97%)	
Unknown/No Response	0 (0.00%)	24 (36.36%)	24 (15.79%)	

	Birth Method Counts (and Percentages)		*Total*	
	*Comparison*	*Caregiving Adversity*		
Vaginal Birth	55 (63.95%)	19 (28.79%)	74 (48.68%)	p < .000*
C-Section	30 (34.88%)	13 (19.70%)	43 (28.29%)	
Unknown/No Response	1 (1.16%)	34 (51.52%)	35 (23.03%)	

	Perinatal Antibiotic Exposure Counts (and Percentages)		*Total*	
	*Comparison*	*Caregiving Adversity*		
Yes	10 (11.63%)	1 (1.52%)	11 (7.24%)	p < .000*
No	64 (74.42%)	14 (21.21%)	78 (51.32%)	
Unknown/No Response	12 I13.95%)	51 (77.27%)	63 (41.45%)	

	Mean Coronavirus Impact^2^ (and Standard Deviation)		*Total*	
	*Comparison*	*Caregiving Adversity*		
Mean Coronavirus Impact (SD)	5.14 (4.00)	4.82 (3.55)	5 (3.80)	p = .602

	Highest Caregiver Education Counts (and Percentages)		*Total*	
	*Comparison*	*Caregiving Adversity*		
Graduate Degree	48 (55.81 %)	31 (46.97%)	79 (51.97%)	p = .217
Bachelor's degree	18 (20.93%)	22 (33.33%)	40 (26.32%)	
Less than Bachelor's	16 (18.60%)	9 (13.64%)	25 (16.45%)	
No Response	4 (4.65%)	4 (6.06%)	8 (5.26%)	

	Siblings in the Study Counts (and Percentages)		*Total*	
	*Comparison*	*Caregiving Adversity*		
Has at least 1 sibling in study	36 (41.86%)	34 (51.52%)	70 (46.05%)	p = .308
Has no siblings in study	50 (58.14%)	32 (48.48%)	82 (53.95%)	

	Mean Waist-to-Height Ratio (and Standard Deviation)		*Total*	
	*Comparison*	*Caregiving Adversity*		
Mean Waist-to-Height Ratio (SD)	0.49 (0.07)	0.47 (0.04)	0.48 (0.07)	p = .136

	Oral Health at 1-Year Follow-Up^3^ Counts (and Percentages)		*Total*	
	*Comparison*	*Caregiving Adversity*		
Any Oral Health Problem	7 (12.96%)	7 (22.58%)	14 (16.47%)	p = .397
No Oral Health Problem	47 (87.04%)	24 (77.42%)	71 (83.53%)	

	Toothbrushing at 1-Year Follow-Up Counts (and Percentages)		*Total*	
	*Comparison*	*Caregiving Adversity*		
Twice or More a Day	30 (69.77%)	17 (65.38%)	47 (68.12%)	p = .049*
Once a Day	7 (16.28%)	9 (34.62%)	16 (23.19%)	
Less than Once a Day	6 (13.95%)	0 (0.00%)	6 (8.70%)	

	Flossing at 1-Year Follow-Up Counts (and Percentages)		*Total*	
	*Comparison*	*Caregiving Adversity*		
Once or More a Day	20 (46.51%)	11 (42.31%)	31 (44.93%)	p = .928
Less than Once a Day	23 (53.49%)	15 (57.69%)	38 (55.07%)	

Table 1 shows counts of categorical variables and means of continuous variables for the Comparison and CA groups, as well as for the complete sample. For continuous variables, standard deviation is shown in parentheses, and for counts, percentages are shown in parentheses within each group. For variables collected at the time of the 1st study visit, values for participants who did not respond or did not know the information are included. For variables collected at 1-year follow-up, only responders are shown. The last column shows p-values for differences between the CA and Comparison groups using Student's T tests for continuous variables and chi squared tests for categorical. ^1^ These categories refer to feeding exclusively with breastmilk, including breastfeeding and feeding breastmilk with a bottle; feeding a combination of breastmilk and formula; feeding exclusively with formula; and either a response that the information was unknown or no response altogether. ^2^ This scale was completed by caregivers and ranged from 0 to 15 (Supplement 3). ^3^ Oral health and oral hygiene information were collected from a subsample of N = 85 participants at 1-year follow-up. Participants were classified as having any oral health problem if they endorsed having problems with bleeding gums, caries/cavities, toothache, or sore gums.

#### 2.1.2 Caregiving adversity

Caregiving adversity was assessed via parent report of the child's caregiving history. To be included in the CA group, participants must have met at least one of the following conditions: been adopted internationally from institutional or foster care; adopted domestically from foster or kinship care; be in guardianship care with a non-biological parent caregiver (kinship care or foster parent); have had extensive separation from a primary caregiver for other reasons (e.g., parental incarceration); and/or have been exposed to significant maltreatment at the hands of a caregiver. This operationalization was designed to examine the effects of caregiving related adversities that cut across specific sociolegal subtypes and were united by virtue of their impact on the caregiver-child relationship. This relationship is ecologically significant and has evolved in mammals to foster the survival and guide the development of youth, making it relevant within humans and across mammalian species (Callaghan et al., 2019). Critically, studies have shown that caregiving related adversities (even when comprised of diverse caregiving experiences), have a unique impact on the brain and behavioral outcomes, as opposed to non-interpersonal adversities, e.g., poverty (Vannucci et al., 2023), justifying our approach to examine diverse CAs together. However, to probe possible effects of CA subtypes, we conducted a supplementary analysis that re-tested significant findings using CA as a multicategorical variable (domestically adopted, internationally adopted, or Comparison; Supplement 1).

Moreover, we chose to study development in CA exposed individuals who were now in stable care arrangements (e.g., adoption, guardianship) because it enables examination of lasting effects of CA that are largely restricted to early life, while lessening the impact on ongoing adversities on study outcomes. In order to better understand the effects of CA duration, we conducted a supplementary analysis that re-tested significant findings within the CA group only, controlling for time since entry into stable care (Supplement 2).

#### 2.1.3 Health outcomes

We used three measures of participants’ health that were proxy reported by their caregivers as outcomes in this study: fatigue subscale on the Pediatric Quality of Life (PedsQL) (Varni et al., 2015), and somatic complaints and internalizing symptoms subscales of the Child Behavior Checklist (CBCL) (Achenbach, 2004).

#### 2.1.4 Covariate selection

The following covariates were selected a priori, based on literature suggesting their lasting associations with the oral microbiome: child's age and sex (Dzidic et al., 2018; Dashper et al., 2019; Kumar, 2013), primary style of infant feeding (Dzidic et al., 2018; Eshriqui et al., 2020) (breastmilk, formula, combination of breastmilk and formula, or unknown), child's birth mode (Dzidic et al., 2018) (vaginal birth, C-section, or unknown), and prenatal or early postnatal antibiotic exposure (Dzidic et al., 2018; Gomez-Arango et al., 2017b) (exposure to antibiotics, no exposure to antibiotics, or exposure unknown). Because many caregivers did not know the child's infant feeding style, birth mode, or perinatal antibiotic exposure history, we included “Unknown” as a category here. We also included caregiver-reported impact of the COVID-19 pandemic on the family as a covariate (see Supplement 3 for details of the scale), given that a large fraction of families enrolled in our study during the pandemic. Only one caregiver reported that their child was not cisgender and no caregivers reported that their child used alcohol (at levels that qualified for study exclusion), thus gender and alcohol use were not included as covariates. Additionally, because our sample was uniformly high in socioeconomic status (assessed by caregiver education; see Table 1), we did not use this variable as a covariate in any analyses, though we did test whether caregiver education was associated with children's hair cortisol in a supplemental analysis (see Supplement 4).

Although oral hygiene behaviors and oral health symptoms were not reported concurrent with the oral microbiome sample, information about these variables was reported approximately 12 months after the samples were collected. Items for two scales assessing oral hygiene behaviors and oral health symptoms were adapted from Simpson et al. (2020) (Table 1, see Supplement 5 for details), and were proxy reported by parents for participants younger than 9 and were self-reported by participants who were 9 years or older. We did not include oral health symptoms as a covariate in any analyses because problems such as caries (Tanner et al., 2016) and gum disease (Hajishengallis, 2014) have been shown to be outcomes of ecological dysregulation of the oral microbiome, rather than a potential confounder of the relationship between CA or cortisol and oral microbiome. In contrast, we suspected that oral hygiene behaviors (e.g., brushing and flossing) likely partially mediated the association between CA and oral microbiome composition, with adversity potentially leading to less effective brushing and flossing (Myran et al., 2023) and this in turn affecting the microbiome (Burcham et al., 2020). Thus, to improve causal inference, we did not control for oral hygiene behaviors in primary analyses involving CA impacts on the microbiome (Wysocki et al., 2022). However, because oral hygiene could potentially confound the effects of cortisol on the oral microbiome, given that oral hygiene can affect the microbiome (Burcham et al., 2020) and could potentially affect cortisol levels (Pani and Al Odhaib, 2013), we included a supplementary analysis to re-test any significant effects of cortisol while controlling for oral hygiene behaviors (see Supplement 6).

#### 2.1.5 Procedure

*In person data collection:* 27 participants completed in-person data collection prior to the shutdown of in-person research activities due to COVID-19. These participants were invited to our research laboratory at UCLA with their caregiver. Under the instruction of trained researchers, they completed behavioral tasks and interviews, were measured for height, weight, and waist circumference, and gave biological samples, including hair and saliva. Caregivers reported the participant's demographic information and completed questionnaires. Participants aged 9 years and older could choose whether to complete questionnaires independently or whether to have the items read to them by a researcher; participants aged 8 years or younger always had the items read to them by a researcher.

*Online data collection:* Due to safety concerns caused by the COVID-19 pandemic, in-person data-collection was halted after the first 26 participants, and the remaining 126 participants completed data collection remotely. These participants and their caregivers received data collection materials by mail. Over Zoom video calls, researchers instructed caregivers and participants on completion of behavioral tasks, height, weight, and waist circumference measurements, and hair and saliva sample collection. Caregivers could choose whether to complete questionnaires and report demographic information on paper or online using the Redcap data collection system. Similar to the in-person data collection, participants 9 years and older could choose whether to complete questionnaires independently using Redcap or whether to have the items read to them by a researcher; participants aged 8 years and younger always had the items read to them by a researcher. See supplementary analysis for comparison of in-person vs. remote participation on primary study outcomes (Supplement 7).

#### 2.1.6 Hair cortisol storage and assay

Tonic stress was measured through hair cortisol (which has been shown to have a correlation as high as 0.61 with the prior 30-day average of salivary cortisol (Short et al., 2016)), assayed from 3 cm hair samples. Under instruction from a research assistant and with help from a caregiver, participants’ hair samples were collected from underneath the crown of the head and cut close to the root. Three samples were stored at −20 Celsius, and were then thawed before processing. The remaining 139 were stored at room temperature. Primary findings involving hair cortisol that were significant at p < .05 and/or q < 0.25 were re-tested in a robustness analysis controlling for hair storage temperature (see Supplement 8). All samples were shipped at ambient temperature to the Meyer lab, where they were processed and analyzed according to the methods described in Meyer et al. (2014) with minor modifications. Briefly, each sample was weighed, washed twice with isopropanol to remove external contaminants, and then air-dried. Washed samples were ground to a fine powder using a bead mill, extracted overnight into methanol, and centrifuged to spin down the beads and the powdered hair. An aliquot of the methanol extract was transferred to a clean tube, dried using a vacuum evaporator, and then reconstituted in assay buffer. Reconstituted extracts were spin-filtered to remove any residual particulate material, then assayed in duplicate along with standards and quality controls using the Arbor Assays DetectX Cortisol ELISA kit. Intra- and inter-assay coefficients of variation for this assay were both <10%. In total, 142 hair samples were collected (63 from the CA group and 79 from the Comparison group), of which all but 1 (from the Comparison group, which was too small to process) were assayed for cortisol levels.

#### 2.1.7 Saliva sample collection, storage, sequencing and pre-processing

Saliva samples were collected via OMNIgene®•ORAL sample collection and stabilization kits (DNA Genotek). After endorsing that they had not consumed any food or beverages for at least 30 min, participants collected the samples under instruction from research assistants and with help from a caregiver. In total, 152 participants (n = 66 from the CA group and n = 86 from the Comparison group) collected saliva samples. One participant (from the Comparison group) collected the sample via a swab, which was an accessory to the OMNIgene kit; the remaining 151 samples were passive drool. Five samples (3 CA, 2 Comparison) were frozen directly in the collection tube. The remaining samples were incubated at 50 °C for 2 h, vortexed, aliquoted into cryogenic tubes and frozen at −20 °C. The samples were shipped to the sequencing site on dry ice to prevent thawing. Primary findings involving the oral microbiome that were significant at p < .05 and/or q < 0.25 were re-tested in a robustness analysis controlling protocol variation (length of sample storage before incubation and whether or not the sample was frozen directly in the collection tube; see Supplement 9).

Amplicon sequencing of the V4 region of the 16S gene was performed with the 515f/806r primer set (Caporaso et al., 2011) following the Earth Microbiome Project (EMP protocol) by Arizona State University lab services. PCR amplifications for each sample were done in duplicate, then pooled and quantified using an accublue kit. A no template control sample was included during the library preparation as a control for extraneous contamination. 240 ng of DNA per sample were pooled and cleaned using QIA quick PCR purification kit (QIAGEN). The pool was quantified by using the qubit. Then, the DNA pool was diluted to a final concentration of 4 nM, then denatured and diluted to a final concentration of 4 pM with a 25% of PhiX. The DNA library was then loaded in the MiSeq Illumina and run using the version 2 module, 2x250 paired-end, following the directions of the manufacturer. Three samples from the CA group were not successfully sequenced on the first run, and were therefore re-run, resulting in successful sequences for those samples.

We pre-processed the raw sequences using Qiime2 software, version 2022.2.1 (Bolyen et al., 2019). Sequences were denoised and amplicon sequence variants (ASVs) inferred using the DADA2 method (Callahan et al., 2016). A phylogenetic tree was constructed using FastTree (Price et al., 2010). Taxonomic assignment was conducted using a bespoke Naïve Bayes classifier trained with a k-mer length of 12 (Bokulich et al., 2018; Kaehler et al., 2019). For calculation of alpha and beta diversity metrics, data were rarefied to 14,697, which was the highest depth at which all samples were retained.

#### 2.1.8 Statistical analyses

Analyses were conducted using R software version 4.3.1.

Unless otherwise stated, any log transformations were performed using the natural log.

We used Welch's t-tests to compare the CA and Comparison groups on the 3 health outcomes (fatigue, somatic complaints, and internalizing symptoms) and on hair cortisol. Welch's t-tests were chosen to avoid statistical assumptions of equal variance between groups. Post-hoc transformations of the health outcome scores were implemented (log, square root, or no transformation, chosen by visual inspection) to reduce skewedness. We calculated 95% confidence intervals (CIs) of the difference in group means.

Using Qiime2, the following alpha diversity metrics were calculated: Faith's phylogenetic diversity (Faith, 1992), count of observed features, Shannon's diversity (Shannon, 1948), and Pielou's evenness (Pielou, 1966). We elected to use these four metrics of richness as they each capture unique information, providing the most comprehensive picture of the microbiome: phylogenetically weighted (Faith's) and unweighted (observed features), evenness of the microbiome (Pielou's), and a combination of richness and evenness (Shannon's). Multiple linear regressions were calculated to correlate each of these metrics with group membership, log-transformed hair cortisol, and their interaction, adjusting for all selected covariates. Semi-partial correlation coefficients (*r*) were calculated for statistically significant results.

Qiime2 was also used to calculate the following beta diversity dissimilarity matrices: Jaccard (1912), Bray-Curtis (Bray and Curtis, 1957), Unweighted UniFrac (Lozupone and Knight, 2005), and Weighted UniFrac (Chang et al., 2011). Using the *adonis 2* function in the *vegan* package (Okansen et al., 2022) for R, we examined how much of the variance in the distance matrices was explained by group membership, log-transformed hair cortisol and their interaction, adjusting for all selected covariates. We used the sequential method (terms are tested sequentially), with the interaction term tested last. Alternative results calculated with the marginal method, which calculates the effect of each term controlling for all other terms, but cannot test main effects, are available in Supplement 10.

Differential abundance was calculated with the MaAslin2 package for R (Mallick et al., 2021) using center log-ratio normalization and filtering for features that were present in at least 20% of samples. Benjamini-Hochberg multiple comparison correction was selected, with a maximum false discovery rate (FDR) of 0.250. This relatively high threshold was selected due to the exploratory nature of the analysis, following published recommendations for biomarker discovery (Aatsinki et al., 2019). The q-statistic associated with each test has been provided in tables in the main text and Supplement 11to enable interpretation of the chance that each significant result is a false positive. Continuous predictor variables were standardized. We examined the association between feature abundance (at each taxonomic level from ASV to phylum), CA group membership, log-transformed hair cortisol and the interaction between CA group membership and log-transformed hair cortisol, adjusting for all selected covariates. Additionally, semi-partial correlation coefficients (*r*) were calculated for statistically significant results.

As a final step, we tested the association between the microbiome and each health outcome: fatigue, somatic complaints, and internalizing symptoms, focusing on microbiome diversity metrics or features that were significantly associated with CA, cortisol, or their interaction. Each of these models adjusted for group membership, log transformed cortisol, the interaction between group membership and cortisol, and all selected covariates, except for beta diversity tests, as the sequential method tests interaction terms last.

## 3 Results

### 3.1 Group differences in health outcomes & cortisol

The CA group scored significantly higher than the Comparison group on the CBCL somatic complaints score (log-transformed; *t* (111.5) = −3.94, p < .000, 95% CI = [−0.90,-0.30]), the CBCL internalizing symptoms score (log-transformed; *t* (121.75) = −4.80, p < .000, 95% CI = [−1.15,-0.48]), and the PEDS-QL fatigue score (square root-transformed; *t* (146.63) = −5.04, p < .000, 95% CI = [−0.43, −0.19]). Hair cortisol, which reflects circulating cortisol levels across the last 1–3 months, did not significantly differ between groups (log-transformed; *t* (138.79) = 0.22, p = .823, 95% CI = [−0.27,0.34]), suggesting that ongoing physiological stress was comparable between the two groups.

#### 3.1.1 Alpha diversity

Adjusting for hair cortisol levels, there were no significant main effects of CA on Faith's diversity (b = −1.72, *t* (127) = −1.86, p = .066), observed features (b = −17.54, *t* (127) = −1.60, p = .112), Pielou's evenness (b = 0.03, *t* (127) = 1.40, p = .165) or Shannon's diversity (b = 0.04, *t* (127) = 0.15, p = .880). Adjusting for CA, there was a significantly negative main effect of hair cortisol on Faith's diversity (b = −0.65, *t* (127) = −2.88, *r* = −0.24, p = .005) and observed features (b = −6.41, *t* (127) = −2.39, *r* = −0.20, p = .018), but not Pielou's evenness (b = 0.01, *t* (127) = 1.54, p = .125) or Shannon's diversity (b = −0.00, *t* (127) = −0.04, p = .965). The interaction between CA group and cortisol was significantly associated with Faith's diversity (b = 0.82, *t* (127) = 2.24, *r* = 0.19, p = .027), which was driven by a negative association between cortisol and Faith's diversity in the Comparison group (b = −0.65, *t* (127) = −2.88, *r* = −0.24, p = .005), but not the CA group (b = 0.17, *t* (127) = 0.59, p = .555). The interaction between CA group and cortisol was not significantly associated with observed feature counts (b = 7.57, *t* (127) = 1.74, p = .085), Pielou's evenness (b = −0.01, *t* (127) = −1.16, p = .248) or Shannon's diversity (b = −0.01, *t* (127) = 0.08, p = .936). Full results are available in Supplement 11.

#### 3.1.2 Beta diversity

Adjusting for hair cortisol, the main effect of CA was small but significant for the Bray-Curtis (R^2^ = 0.01, F = 2.06, p = .016), Weighted UniFrac (R^2^ = 0.02, F = 2.30, p = .047), and Jaccard (R^2^ = 0.01, F = 1.47, p = .025) dissimilarity indices (see Fig. 3a–c), but not Unweighted UniFrac (R^2^ = 0.01, F = 1.29, p = .183). Adjusting for CA exposure, there was no significant main effect of cortisol for the Bray-Curtis (R^2^ = 0.01, F = 0.91, p = .501), Jaccard (R^2^ = 0.01, F = 1.22, p = .110), Unweighted UniFrac (R^2^ = 0.01, F = 1.08, p = .341), or Weighted UniFrac (R^2^ = 0.01, F = 1.93, p = .117) indices. The interaction of CA group and cortisol was significantly associated with Jaccard dissimilarity (R^2^ = 0.01, F = 1.33, p = .039, see Fig. 3d), was not significant but was trending for Unweighted UniFrac dissimilarity (R^2^ = 0.01, F = 1.67, p = .055; see Fig. 3e), and was not significant for Bray-Curtis dissimilarity (R^2^ = 0.01, F = 1.21, p = .255) and for Weighted UniFrac dissimilarity (R^2^ = 0.01, F = 0.94, p = .427). Full results of all 4 models are available in Supplement 11.

**Fig. 1:**
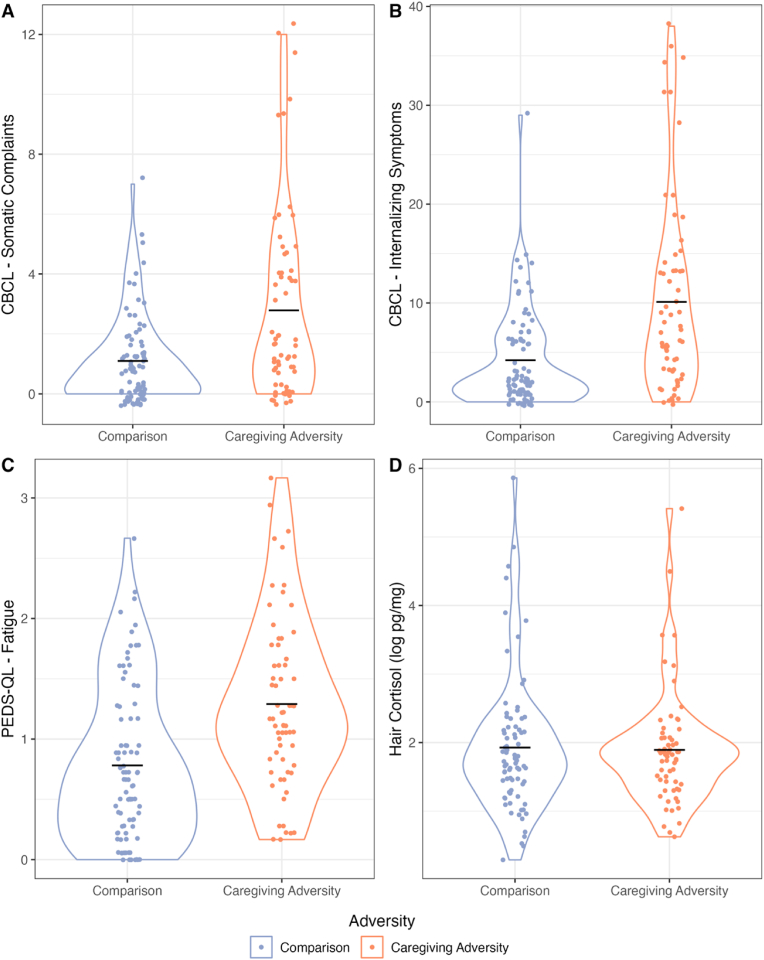
Fig. 1a–d shows group differences in health outcomes (CBCL somatic complaints, CBCL internalizing symptoms, and PEDS-QL Fatigue) and cortisol. Points showing Comparison group members are in blue and CA group members are in orange. Points are jittered along the X axis. The violin plots show the distribution of the abundance of each respective health outcome or log-transformed cortisol in each group, with greater width showing greater density of observations at each point in the Y axis. A black, horizontal line shows the mean for each group. (For interpretation of the references to colour in this figure legend, the reader is referred to the Web version of this article.)

**Fig. 2:**
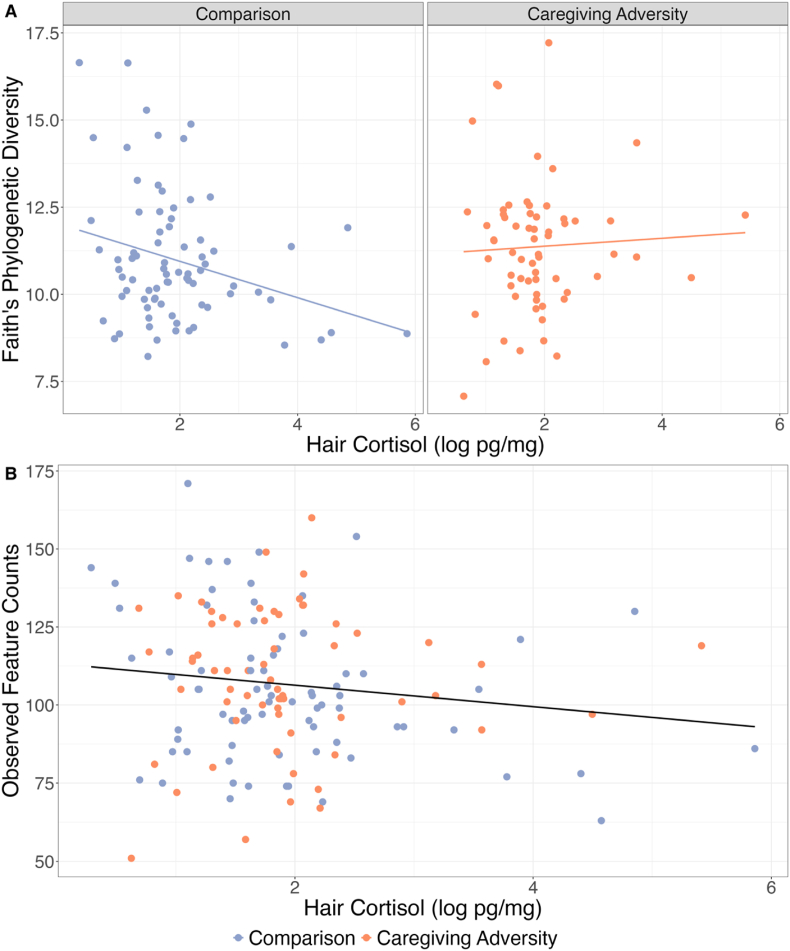
Fig. 2a and b shows log-transformed values of hair cortisol (pg/mg) on the X axes and alpha diversity metrics on the Y axes (Faith's diversity in plot 2a, observed feature counts in plot 2b). The left facet of 2a shows values for participants in the Comparison group (blue) and the right facet shows the Caregiving Adversity group (orange). In 2a, a line shows the simple slope of the respective alpha diversity metric over log-transformed cortisol in each group. In 2b, a black line shows the slope across both groups. The main effect of cortisol was significantly negative for both Faith's diversity and observed feature counts. The interaction between CA and cortisol was significantly associated with Faith's diversity, shown in plot 2a, and driven by a negative association between cortisol and diversity in the comparison, but not CA group. (For interpretation of the references to colour in this figure legend, the reader is referred to the Web version of this article.)

**Fig. 3:**
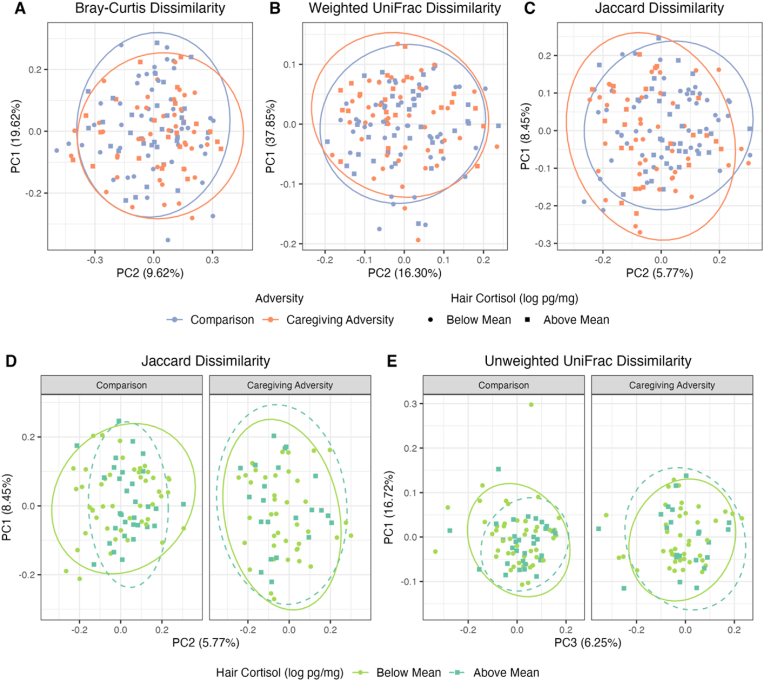
Fig. 3a–e shows principal coordinates plots of beta diversity dissimilarities (Bray-Curtis in plot 3a, Weighted UniFrac in plot 3b, Jaccard in plots 3c and 3d, and Unweighted UniFrac in plot 3e). The X and Y axes show the principal coordinates that explained the most and second most variance, respectively, in each dissimilarity metric in 3a-3d; for Fig. 3e, the first and third principal coordinates were selected to improve visibility of the data. The amount of variance explained is shown in the axis labels. Ellipses show a 95% confidence level for a multivariate t distribution for participants in each CA group in plots 3a-3c, and for participants with log-transformed cortisol values below the sample mean (green, solid line) or above the mean (blue, dotted line) in plots 3d-3e. Similarly, log-transformed cortisol values below the mean are represented by circles and those above the mean with squares. Plots 3d-3e are faceted such that participants in the Comparison group are shown on the left, and Caregiving Adversity group on the right. (For interpretation of the references to colour in this figure legend, the reader is referred to the Web version of this article.)

**Fig. 4:**
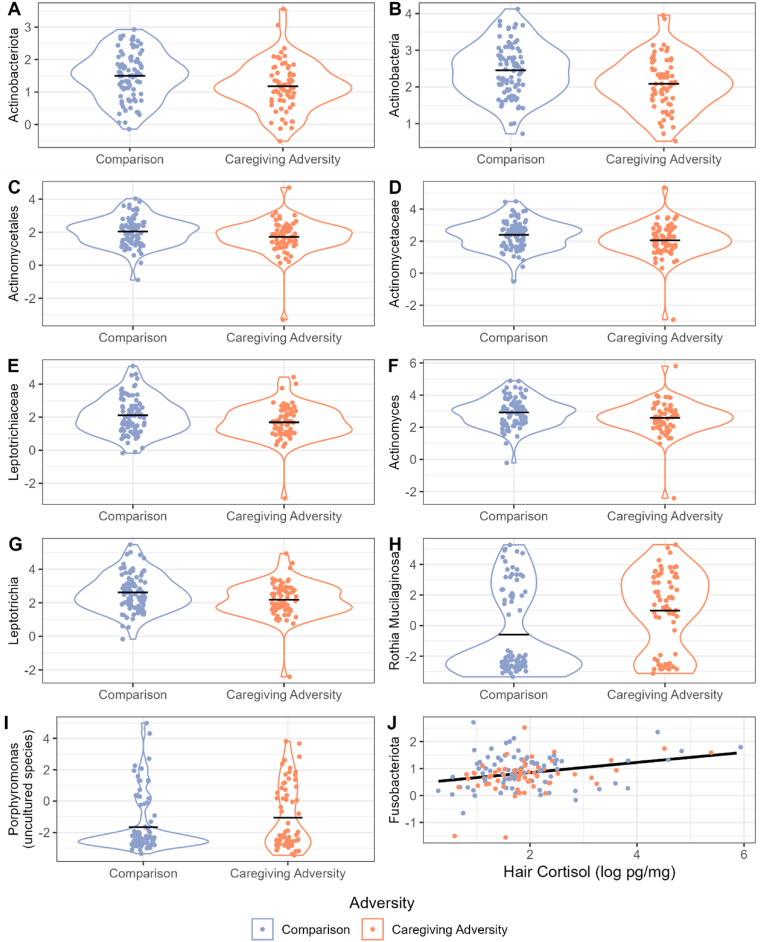
Fig. 4a–j *shows abundance of selected taxa (*Actinobacteriota *in 4a,* Actinobacteria *in 4b,* Actinomycetales *in 4c,* Actinomycetaceae *in 4d,* Leptotrichiaceae *in 4e,* Actinomyces *in 4f*, Leptotrichia *in 4g,* R. Mucilaginosa *in 4h, an uncultured* Porphyromonas *species in 4i, and* Fusobacteriota *in 4j). The Y axis of each plot shows abundance of each taxon after filtering and transformation (center log ratio; CLR), as described in the Methods section. The X axes of 4a-4i show group membership. The X axis of*Fig. 4j *shows the log of pg/mg of hair cortisol. Points showing Comparison group members are in blue and CA group members are in orange. Points in*Fig. 4a–i *are jittered along the X axis.*Fig. 4a–i *are violin plots that show the distribution of the abundance of each respective taxon in each group, with greater width showing greater density of observations at each point in the Y axis. The distributions of the Comparison group are shown in blue and the CA group in orange. A black, horizontal line shows the mean for each group.*Fig. 4j *includes a black line that shows the slope of* Fusobacteriota *abundance over hair cortisol values*. (For interpretation of the references to colour in this figure legend, the reader is referred to the Web version of this article.)

**Fig. 5:**
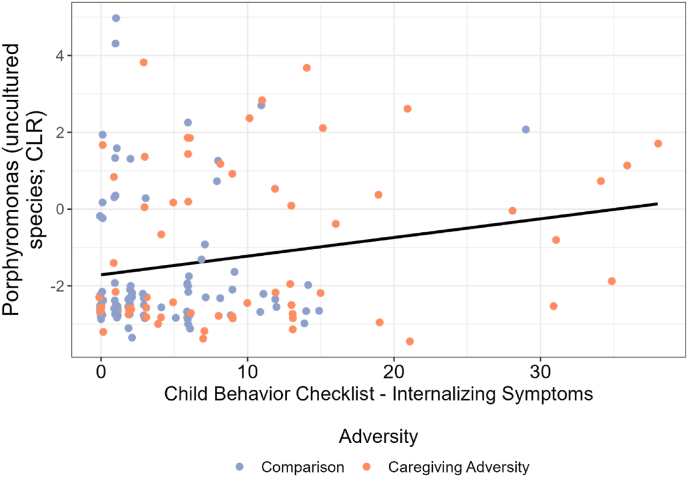
Fig. 5 shows abundance of an ASV identified as an uncultured *Porphyromonas* species. The Y axis shows abundance of this taxon after filtering and transformation, as described in the Methods section. The X axis shows internalizing symptoms. Points showing Comparison group members are in blue and CA group members are in orange. A black line shows the slope of ASV abundance over internalizing symptoms. (For interpretation of the references to colour in this figure legend, the reader is referred to the Web version of this article.)

To probe the interaction of CA and cortisol in explaining variance in Jaccard dissimilarity, we conducted a post-hoc analysis wherein we tested whether cortisol explained significant variance in Jaccard dissimilarity independently in each of the groups, controlling for the same covariates as the original analysis. Cortisol explained variance in Jaccard dissimilarity in the Comparison group (R^2^ = 0.02, F = 1.58, p = .006) but not the CA group (R^2^ = 0.02, F = 1.10, p = .255).

#### 3.1.3 Differential abundance

We used the MaAslin2 package in R to test if CA group membership, hair cortisol, or their interaction was associated with the differential abundance of taxa, adjusting for all covariates. At the phylum level, CA was negatively associated with *Actinobacteriota* (b = −0.56, q = 0.172, *r* = −0.20)*.* CA was also negatively associated with *Actinobacteria* at the class level (b = −0.68 q = 0.083, *r* = −0.26), *Actinomycetales* at the order level (b = −1.03, q = 0.056, *r* = −0.31), *Actinomycetaceae* (b = −1.07, q = 0.056, *r* = −0.30) and *Leptotrichiaceae* (b = −1.12, q = 0.113, *r* = −0.28) at the family level, and *Actinomyces* (b = −1.02, q = 0.076, *r* = −0.30) and *Leptotrichia* (b = −1.10, q = 0.139, *r* = −0.28) at the genus level. At the ASV level, CA was positively associated with a feature identified as *Rothia Mucilaginosa* (b = 2.93, q = 0.138, *r* = 0.29) and a feature identified as an uncultured *Porphyromonas* (b = 1.94, q = 0.194, *r* = 0.27). Cortisol was positively associated with the phylum *Fusobacteriota* (b = 0.15, q = 0.230, *r* = 0.19). The interaction between CA and hair cortisol was not significantly associated with any features. Full results are available in Supplement 11.

#### 3.1.4 Health outcomes

We explored whether any of the alpha diversity, beta diversity, or taxonomic abundance metrics identified in the previous section as being significantly associated with CA, cortisol, or the interaction of CA and cortisol were associated with any of the following 3 health outcomes: fatigue, somatic complaints, or internalizing symptoms, when adjusting for the variance associated with CA, cortisol and their interaction.

Neither alpha diversity nor beta dissimilarity indices were associated with any of the health outcomes (see Supplement 11). One target microbiota differential abundance metric, the uncultured *Porphyromonas* ASV, which was higher in the CA than comparison group, was significantly positively associated with a health outcome, internalizing symptoms (b = 0.34, q = 0.159, *r* = 0.16). Full results are available in Supplement 11.

## 4 Discussion

This study examined the oral microbiomes of children and adolescents exposed to caregiving adversity (CA) and tested whether the experience of caregiving adversity moderated associations between cortisol and the oral microbiome. We found that when controlling for its interaction with hair cortisol, the main effect of CA group membership had no significant association with alpha diversity; explained a small amount of variance in beta diversity; and was significantly associated with a range of differentially abundant taxa, mostly in the *Actinobacteriota* phylum (as discussed below). In contrast, when controlling for its interaction with CA group membership, the main effect of hair cortisol was negatively associated with two alpha diversity richness measures, Faith's diversity and observed feature counts (driven by the comparison group), but it was not significantly associated with any beta diversity metrics. This suggests that cumulative exposure to high cortisol levels over several weeks may alter the composition of the oral microbiome, leading to decreased richness. Additionally, hair cortisol was positively associated with abundance of the phylum *Fusobacteriota*. Interestingly, we also observed that the interaction between CA group membership and cortisol was associated with Faith's diversity, such that a significantly negative association between hair cortisol and diversity was observed in the comparison group, but this association was blunted in the CA group. This interaction term also explained significant (but small) variance in Jaccard dissimilarity (beta diversity), though it was not associated with any differentially abundant microbes. Together these data suggest that CAs from earlier in childhood have a lasting impact on the oral microbiome throughout later childhood and adolescence, and that this effect may be realized, in part, via CA exposure altering the relationship between recent physiological stress (cortisol) and the oral microbiome.

The fact that different relationships were seen between cortisol and oral microbiome composition in CA and comparison groups was a particularly interesting finding in this study. In terms of alpha diversity, this association suggests that cortisol may regulate the richness of the oral microbiome, but that experience with early caregiving adversity may disrupt that regulatory association. Similarly, cortisol was associated with greater variance in the Jaccard dissimilarity index for samples within the Comparison group, but not for samples within the CA group. Although this was a small effect, the fact that this interaction term significantly explained variance in both alpha and beta diversity metrics indicates a degree of robustness. Together, these effects support the possibility that microbes of the oral cavity acquire cortisol insensitivity in hosts with 10.13039/100004676CA exposure. Indeed, as mentioned in the introduction, several types of host tissue have been shown to exhibit cortisol insensitivity following CA (McGowan et al., 2009; Tyrka et al., 2012), suggesting that the microbiome could display a similarly altered cortisol response in CA-exposed hosts. Such cortisol insensitivity could be due to changes in taxonomic composition, that is, microbes that are less sensitive to cortisol may become more prevalent in the community within CA-exposed hosts. It is also possible that the association between hair cortisol and oral microbiome diversity in the Comparison group may not be causal, but due to a confounder that affects both variables. For example, sleep problems may lead to altered levels of cortisol (Kuhlman et al., 2020b) and of certain oral bacteria (Liu et al., 2020). Longitudinal and experimental data (e.g., in rodent studies or human intervention studies) are necessary to reveal more information about the causal nature of the relationship between cortisol and the oral microbiome.

While the interaction between CA and cortisol was not significantly associated with any differentially abundant microbes, several taxa were associated with CA or cortisol independently. In other words, both past adversities and recent stress were associated with unique features of the microbiome. Starting with recent stress, hair cortisol, reflecting accumulations across the past several months, was modestly positively associated with one taxa - the phylum *Fusobacteriota*, which contains both commensal and pathogenic members. Critically, *Fusobacteriota* has been shown to increase its gene transcription in response to cortisol in vitro (Duran-Pinedo et al., 2018), bolstering the idea that it is intimately regulated by cortisol levels. In terms of past adversity exposure, we also observed associations with several taxa within the *Fusobacteriota* phylum. Specifically, the CA group had a lower abundance of the genus *Leptotrichia*. Interestingly, some members of the *Leptotrichia* genus are also highly responsive to cortisol in terms of their transcriptomic activity (Duran-Pinedo et al., 2018). Thus, the depletion of this genus in the 10.13039/100004676CA group could broadly support the view that 10.13039/100004676CA might reduce the abundance of microbes that are responsive to cortisol. However, some strains of *Leptotrichia* have also been associated with higher internalizing symptoms in adolescents (Simpson et al., 2020), and in this study, the CA group had higher rates of internalizing symptoms but lower *Leptotrichia* than non-CA exposed youth (Conway et al., 2018). Also depleted in the CA group were *Actinobacteriota*, which are among the most common phyla of the oral microbiome (Sedghi et al., 2021). Similarly to *Leptotrichia*, *Actinobacteriota* may be responsive to cortisol. For example, abundance of *Actinobacteriota* has been positively associated with basal salivary cortisol in a sample of typically-developing adolescents (Simpson et al., 2020). While the functional capacities of taxa within this phylum are tremendously variable, these data, in combination with the current findings, also support a possible depletive effect of 10.13039/100004676CA exposure on cortisol-responsive bacteria.

At a higher taxonomic resolution, more of our findings were congruent with the elevated oral health risk previously identified in CA youth (Sarvas et al., 2021). Specifically, within the *Actinomycetaceae* family, *Actinomyces*, a genus which includes strains thought to inhibit oral pathogen growth (Sedghi et al., 2021), was decreased in the CA group. Indeed, the abundance of *Actinomyces* is thought to be increased by periodontal health interventions (Zhang et al., 2021), suggesting that its depletion in the CA group may indicate risk for poor oral health. *Actinomyces* is also thought to be an important nitrate-reducer, a function which may help maintain healthy blood pressure (Sato-Suzuki et al., 2020). Nonetheless, *Actinomyces* depletion in the CA group may not be entirely harmful: *Actinomyces* also degrades carbohydrates, which can lead to an environment more conducive to caries (Sedghi et al., 2021), especially in combination with diet.

The health associations of differentially abundant microbes remained mixed even at the highest level of resolution (the ASV level). CA was positively associated with two ASVs: *R. mucilaginosa*, a commensal member of the *Rothia* genus, and an uncultured feature belonging to the *Porphyromonas* genus. In past studies, *R. mucilaginosa* interacted with adolescents’ basal cortisol levels to explain variance in anxiety – under low basal cortisol this taxon was positively associated with anxiety, but this association was not significant under high basal cortisol (Simpson et al., 2020). However, in our sample, *R. mucilaginosa*, while significantly associated with CA, was not associated with cortisol nor mental health; more research should examine how consistently this taxon is related to CA, cortisol levels, and mental health. *Porphyromonas*, the genus containing the second ASV that was higher in the CA than comparison group, is best-known for its member species: *Porphyromonas gingivalis,* a keystone pathogen. However, even here the interpretation is not clear-cut, as previous studies have noted that the *Porphyromonas* genus contains both beneficial and highly pathogenic species (reviewed by Guilloux et al. (2021)). As such, more detailed, strain-level information, such as that gained through shotgun metagenomic sequencing, is needed to interpret the association between CA and this taxon. Critically, the association between CA and both of these ASVs was modest. However, considering the variability within our sample in terms of the type of CA experienced, time since CA exposure, and nature of the caregiving environment following CA exposure, these effect sizes are not unexpected and may indicate a remarkably robust effect of CA in the face of such individual differences. More granular sequencing methods, such as shotgun metagenomics, will further elucidate differences at the strain level and provide insights into genetic potential for metabolic functions within the microbiome, which may be more informative than taxonomic information alone.

Amongst the significantly differentially abundant microbes associated with adversity and cortisol in this study, we observed very limited associations with health. Specifically, we saw a small positive association between the relative abundance of the ASV identified as an uncultured member of the *Porphyromonas* genus (which was higher in the CA group) and internalizing symptoms. While there are a number of possible explanations for this association, we speculate that it could be due to the association that members of the *Porphyromonas* genus (reviewed by Hajishengalis et al. (Hajishengallis, 2015)), and internalizing symptoms (Slopen et al., 2013b) share with the third variable of elevated inflammation. In support of this third variable hypothesis, 10.13039/100004676CA has previously been linked to elevated inflammation in youth (Kuhlman et al., 2020a), and in this study, 10.13039/100004676CA is associated with elevated internalizing symptoms and higher *Porphyromonas.* Although members of the *Porphyromonas* genus have been linked to inflammation in adults (Hajishengallis, 2015), there is limited evidence for this effect in youth. As such, future studies incorporating measures of circulating or local inflammatory markers alongside the oral microbiome will provide important mechanistic insights into the links between CA exposure, *Porphyromonas* abundance, and internalizing symptoms.

While the links between microbiome community composition and taxa with behavior were modest in this study, more associations may be revealed when looking at microbiome functional potential, rather than taxonomic associations, and when examining objective, rather than self-reported, health outcomes. Additionally, as oral microbiome dysregulation is typically less severe in youth than in adults, a link between microbial composition and health may be less detectable in the current study design due to the young age of the participants.

Considering the variance in physiological and psychological states associated with adversity, and the fact that youth in this study were relatively healthy overall, the fact that even small associations between CA and microbiome features, and between microbiome features and health is notable. Moreover, the fact that adversity moderated a previously reported association between cortisol and the oral microbiome suggests that a more comprehensive approach to the study of the oral microbiome, that tests for many potential mediators, is warranted. Health behaviors, such as oral hygiene or diet, are especially important to consider. Current or past barriers to accessing dental care could affect the composition of the oral microbiome in CA exposed youth. Physiological variables, such as hypothalamic-pituitary-adrenal (HPA) axis dysregulation or increased inflammation, are also putative mechanisms that need to be accounted for. Longitudinal follow-up of this cohort may reveal additional insight into such mechanisms, with both physiological and behavioral mechanisms considered in tandem. Research into other types of adversity, such as abuse or neglect not resulting in permanent removal from the caregiver, will yield additional information about which characteristics of adversity most impact the oral microbiome. For example, changes in nutrition and the physical environment that a child experiences when transitioning from a birth family to an adoptive family could play an important role in microbiome development, but such an effect is not testable in the current study design.

### 4.1 Limitations

Given the sequencing methods we used, which limited our understanding to taxonomic information, the functional meaning of microbiome differences between the CA and Comparison groups are not knowable in this study. Whole genome metagenomic sequencing, which provides strain-level taxonomic information, as well as information about the functional capacity of the microbial genes should be considered in future samples. Additionally, given the cross-sectional nature of these data, it is unclear how stable individual differences in the microbiome are across time. Longitudinal tracking of this cohort will reveal not only the stability of the group differences in microbiome composition over time, but also additional information about the mechanisms behind these differences, temporal precedence and causal implications, and implications for health and well-being.

Delineating both physiological and behavioral factors contributing to oral microbiome composition is of particular interest, as these may inform different treatments to promote a healthier microbiome. For example, prospective tracking of biomarkers, such as cortisol, behavioral factors such as diet or antibiotic use, and oral health and mental health outcomes would be useful for identifying potential oral microbiome-based treatments. Furthermore, because our sample had relatively high socioeconomic status, with most participants having at least one parent with a bachelor's or graduate degree, we were unable to investigate the effects of socioeconomic status, which has also been shown to moderate the association between cortisol and oral microbiome composition (Boyce et al., 2010).

The nature of this study is primarily exploratory, which was reflected in our use of an exploratory q-value threshold of 0.25 for differential abundance analyses. Due to a relative scarcity of work in this area, we elected to use this exploratory threshold in which we were willing to tolerate up to 25% of our significant differential abundance findings might be type I errors. To aid in interpretation of our findings, we provided exact q-values for all of our significant results, which can be interpreted at the likelihood that the particular test was a false positive, and ranges among our results from 0.056 to 0.230. As our understanding of the relationship between CA and the oral microbiome improves, more targeted methods that allow for fewer type I errors while maintaining statistical power to identify meaningful associations will be possible.

### 4.2 Conclusions

The results of this study demonstrate that, while Comparison youth exhibit a negative association between microbial richness and hair cortisol levels, this association is blunted in CA-exposed youth. These findings suggest that recent stress may affect the oral microbiome of youth, but that a history of exposure to Caregiving Adversity may alter that relationship. Additionally, several microorganisms were differentially abundant in CA-exposed youth, including depletion of several microorganisms identified in other literature as being responsive to cortisol, as well as increased abundance of potential pathogens. Thus, both recent stress and history of CA are important considerations for researchers and clinicians who work with the oral microbiome.

## Code availability

Scripts used to manipulate and analyze data for this manuscript are available at **https://github.com/ngancz/oral_microbiome_caregiving_adversity**.

## CRediT authorship contribution statement

**Naomi N. Gancz:** Conceptualization, Data curation, Formal analysis, Funding acquisition, Investigation, Methodology, Visualization, Writing – original draft. **Francesca R. Querdasi:** Conceptualization, Data curation, Funding acquisition, Investigation, Methodology, Writing – review & editing. **Kristen A. Chu:** Data curation, Investigation, Methodology, Project administration. **Emily Towner:** Data curation, Investigation, Methodology, Project administration, Writing – review & editing. **Eason Taylor:** Data curation, Investigation. **Bridget L. Callaghan:** Conceptualization, Formal analysis, Funding acquisition, Methodology, Project administration, Supervision, Writing – review & editing.

## Declaration of competing interest

The authors declare that they have no known competing financial interests or personal relationships that could have appeared to influence the work reported in this paper.

## Data Availability

Contributing author only - not involved with data management or access
