# Supplementary material for: Characteristics of the oral microbiome in youth exposed to caregiving adversity
Source: Brain Behav Immun Health. 2024 Aug 28;41:100850. doi: 10.1016/j.bbih.2024.100850 (PMC11401114; doi:10.1016/j.bbih.2024.100850)
Supplement: Multimedia component 1 [file mmc1.docx]

Note: differential abundance findings for supplementary analyses are provided in a separate Excel document.

**Supplement 1 – CA subgroups**

**Supplementary Table S1.1**

Association between the interaction between cortisol and CA subgroups and Faith’s Phylogenetic Diversity.

| **term** | **estimate** | **std_error** | **statistic** | **p_value** | **lower_ci** | **upper_ci** |
| --- | --- | --- | --- | --- | --- | --- |
| intercept | 10.331 | 0.962 | 10.741 | 0.000 | 8.427 | 12.235 |
| child_age_years | 0.078 | 0.050 | 1.554 | 0.123 | -0.021 | 0.178 |
| child_sex: male | 0.236 | 0.337 | 0.700 | 0.485 | -0.431 | 0.903 |
| breastfeeding: combination | 0.267 | 0.492 | 0.544 | 0.588 | -0.706 | 1.241 |
| breastfeeding: DK or NA | 0.967 | 0.830 | 1.165 | 0.246 | -0.676 | 2.610 |
| breastfeeding: formula | 0.907 | 0.651 | 1.393 | 0.166 | -0.382 | 2.196 |
| c_section: No | -0.093 | 0.594 | -0.157 | 0.876 | -1.268 | 1.082 |
| c_section: Yes | 0.290 | 0.623 | 0.465 | 0.643 | -0.944 | 1.524 |
| antibiotics: No | 0.706 | 0.509 | 1.387 | 0.168 | -0.302 | 1.713 |
| antibiotics: Yes | 0.709 | 0.774 | 0.916 | 0.361 | -0.823 | 2.242 |
| covid_impact | 0.023 | 0.044 | 0.514 | 0.608 | -0.065 | 0.110 |
| CA Subgroup: domestic | -1.899 | 0.965 | -1.968 | 0.051 | -3.809 | 0.011 |
| CA Subgroup: international | 0.965 | 1.778 | 0.543 | 0.588 | -2.555 | 4.484 |
| log_cortisol | -0.644 | 0.225 | -2.862 | 0.005 | -1.090 | -0.199 |
| CA Subgroup: domestic:log_cortisol | 1.014 | 0.381 | 2.659 | 0.009 | 0.259 | 1.769 |
| CA Subgroup: international:log_cortisol | -0.222 | 0.899 | -0.247 | 0.805 | -2.001 | 1.557 |

**Supplementary Table S1.2**

Association between the interaction between cortisol and CA subgroups and observed feature counts.

| **term** | **estimate** | **std_error** | **statistic** | **p_value** | **lower_ci** | **upper_ci** |
| --- | --- | --- | --- | --- | --- | --- |
| intercept | 83.254 | 11.420 | 7.290 | 0.000 | 60.648 | 105.860 |
| child_age_years | 1.382 | 0.597 | 2.315 | 0.022 | 0.200 | 2.564 |
| child_sex: male | 2.472 | 4.000 | 0.618 | 0.538 | -5.445 | 10.389 |
| breastfeeding: combination | -3.807 | 5.839 | -0.652 | 0.516 | -15.365 | 7.750 |
| breastfeeding: DK or NA | 17.669 | 9.857 | 1.792 | 0.076 | -1.844 | 37.181 |
| breastfeeding: formula | 11.707 | 7.730 | 1.515 | 0.132 | -3.594 | 27.008 |
| c_section: No | 6.040 | 7.051 | 0.857 | 0.393 | -7.918 | 19.997 |
| c_section: Yes | 8.889 | 7.403 | 1.201 | 0.232 | -5.764 | 23.542 |
| antibiotics: No | 9.946 | 6.044 | 1.646 | 0.102 | -2.018 | 21.910 |
| antibiotics: Yes | 14.713 | 9.194 | 1.600 | 0.112 | -3.486 | 32.912 |
| covid_impact | 0.342 | 0.524 | 0.653 | 0.515 | -0.695 | 1.380 |
| CA Subgroup: domestic | -17.270 | 11.459 | -1.507 | 0.134 | -39.953 | 5.413 |
| CA Subgroup: international | 4.139 | 21.111 | 0.196 | 0.845 | -37.649 | 45.927 |
| log_cortisol | -6.387 | 2.673 | -2.390 | 0.018 | -11.678 | -1.097 |
| CA Subgroup: domestic:log_cortisol | 8.987 | 4.529 | 1.985 | 0.049 | 0.023 | 17.951 |
| CA Subgroup: international:log_cortisol | -1.347 | 10.672 | 0.126 | 0.900 | -19.777 | 22.470 |
|  |  |  |  |  |  |  |
|  |  |  |  |  |  |  |

**Supplementary Table S1.3**

Association between the interaction between cortisol and CA subgroups and Jaccard dissimilarity.

|  | **Df** | **SumOfSqs** | **R2** | **F** | **Pr(>F)** |
| --- | --- | --- | --- | --- | --- |
| child_age_years | 1 | 0.444 | 0.016 | 2.216 | 0.001 |
| child_sex | 1 | 0.194 | 0.007 | 0.971 | 0.530 |
| breastfeeding | 3 | 0.731 | 0.026 | 1.217 | 0.027 |
| c_section | 2 | 0.478 | 0.017 | 1.193 | 0.078 |
| antibiotics | 2 | 0.438 | 0.015 | 1.095 | 0.197 |
| covid_impact | 1 | 0.214 | 0.008 | 1.069 | 0.294 |
| CA_subgroup | 2 | 0.531 | 0.019 | 1.326 | 0.015 |
| log_cortisol | 1 | 0.235 | 0.008 | 1.175 | 0.123 |
| CA_subgroup:log_cortisol | 2 | 0.495 | 0.017 | 1.236 | 0.046 |
| Residual | 123 | 24.619 | 0.868 | NA | NA |
| Total | 138 | 28.378 | 1.000 | NA | NA |

**Supplementary Table S1.4**

Association between the interaction between cortisol and CA subgroups and unweighted Unifrac dissimilarity.

|  | **Df** | **SumOfSqs** | **R2** | **F** | **Pr(>F)** |
| --- | --- | --- | --- | --- | --- |
| child_age_years | 1 | 0.137 | 0.017 | 2.370 | 0.007 |
| child_sex | 1 | 0.051 | 0.006 | 0.879 | 0.564 |
| breastfeeding | 3 | 0.187 | 0.023 | 1.078 | 0.301 |
| c_section | 2 | 0.102 | 0.013 | 0.885 | 0.646 |
| antibiotics | 2 | 0.132 | 0.016 | 1.144 | 0.237 |
| covid_impact | 1 | 0.041 | 0.005 | 0.719 | 0.774 |
| CA_subgroup | 2 | 0.144 | 0.018 | 1.247 | 0.161 |
| log_cortisol | 1 | 0.061 | 0.007 | 1.052 | 0.360 |
| CA_subgroup:log_cortisol | 2 | 0.209 | 0.026 | 1.814 | 0.015 |
| Residual | 123 | 7.099 | 0.870 | NA | NA |
| Total | 138 | 8.163 | 1.000 | NA | NA |
|  |  |  |  |  |  |

**Supplementary Table S1.5**

Association between the interaction between cortisol and CA subgroups and Bray-Curtis dissimilarity.

|  | **Df** | **SumOfSqs** | **R2** | **F** | **Pr(>F)** |
| --- | --- | --- | --- | --- | --- |
| child_age_years | 1 | 0.265 | 0.013 | 1.775 | 0.037 |
| child_sex | 1 | 0.148 | 0.007 | 0.992 | 0.424 |
| breastfeeding | 3 | 0.498 | 0.024 | 1.111 | 0.257 |
| c_section | 2 | 0.373 | 0.018 | 1.247 | 0.167 |
| antibiotics | 2 | 0.423 | 0.020 | 1.414 | 0.092 |
| covid_impact | 1 | 0.173 | 0.008 | 1.154 | 0.278 |
| CA_subgroup | 2 | 0.481 | 0.023 | 1.608 | 0.032 |
| log_cortisol | 1 | 0.131 | 0.006 | 0.873 | 0.550 |
| CA_subgroup:log_cortisol | 2 | 0.303 | 0.014 | 1.013 | 0.398 |
| Residual | 123 | 18.390 | 0.868 | NA | NA |
| Total | 138 | 21.184 | 1.000 | NA | NA |

**Supplementary Table S1.6**

Association between the interaction between cortisol and CA subgroups and weighted Unifrac dissimilarity.

|  | **Df** | **SumOfSqs** | **R2** | **F** | **Pr(>F)** |
| --- | --- | --- | --- | --- | --- |
| child_age_years | 1 | 0.032 | 0.012 | 1.638 | 0.145 |
| child_sex | 1 | 0.013 | 0.005 | 0.671 | 0.585 |
| breastfeeding | 3 | 0.043 | 0.015 | 0.728 | 0.707 |
| c_section | 2 | 0.074 | 0.027 | 1.895 | 0.064 |
| antibiotics | 2 | 0.051 | 0.018 | 1.305 | 0.255 |
| covid_impact | 1 | 0.034 | 0.012 | 1.751 | 0.115 |
| CA_subgroup | 2 | 0.058 | 0.021 | 1.477 | 0.155 |
| log_cortisol | 1 | 0.033 | 0.012 | 1.695 | 0.147 |
| CA_subgroup:log_cortisol | 2 | 0.028 | 0.010 | 0.715 | 0.682 |
| Residual | 123 | 2.399 | 0.868 | NA | NA |
| Total | 138 | 2.764 | 1.000 | NA | NA |

**Supplement 2 – Time since entry into stable care**

*Note:* The following analyses are conducted only in the CA group. Instead of a CA by cortisol interaction, these analyses test for main effects of cortisol and time since entry into stable care, as well as all the covariates used in the primary analyses.

**Supplementary Table S2.1**

Association between time since entry into stable care and Faith’s Phylogenetic Diversity.

| **term** | **estimate** | **std_error** | **statistic** | **p_value** | **lower_ci** | **upper_ci** |
| --- | --- | --- | --- | --- | --- | --- |
| intercept | 10.332 | 1.852 | 5.579 | 0.000 | 6.602 | 14.062 |
| child_age_years | 0.198 | 0.129 | 1.536 | 0.132 | -0.062 | 0.458 |
| child_sex: male | -0.609 | 0.568 | -1.071 | 0.290 | -1.754 | 0.536 |
| breastfeeding: combination | -0.049 | 2.261 | -0.022 | 0.983 | -4.603 | 4.504 |
| breastfeeding: DK or NA | -0.765 | 1.491 | -0.513 | 0.610 | -3.767 | 2.238 |
| breastfeeding: formula | -0.882 | 1.336 | -0.660 | 0.512 | -3.573 | 1.809 |
| c_section: No | -0.498 | 0.711 | -0.701 | 0.487 | -1.930 | 0.934 |
| c_section: Yes | 0.686 | 0.918 | 0.747 | 0.459 | -1.163 | 2.534 |
| antibiotics: No | -0.367 | 0.949 | -0.387 | 0.701 | -2.278 | 1.544 |
| antibiotics: Yes | -1.770 | 2.549 | -0.694 | 0.491 | -6.904 | 3.364 |
| covid_impact | 0.146 | 0.079 | 1.852 | 0.071 | -0.013 | 0.305 |
| log_cortisol | 0.001 | 0.313 | 0.004 | 0.997 | -0.630 | 0.633 |
| time_since_stable_care | -0.082 | 0.087 | -0.942 | 0.351 | -0.256 | 0.093 |

**Supplementary Table S2.2**

Association between time since entry into stable care and observed feature counts.

| **term** | **estimate** | **std_error** | **statistic** | **p_value** | **lower_ci** | **upper_ci** |
| --- | --- | --- | --- | --- | --- | --- |
| intercept | 78.739 | 20.376 | 3.864 | 0.000 | 37.699 | 119.778 |
| child_age_years | 3.531 | 1.420 | 2.486 | 0.017 | 0.670 | 6.391 |
| child_sex: male | -3.004 | 6.254 | -0.480 | 0.633 | -15.600 | 9.593 |
| breastfeeding: combination | -25.633 | 24.876 | -1.030 | 0.308 | -75.735 | 24.469 |
| breastfeeding: DK or NA | -5.789 | 16.402 | -0.353 | 0.726 | -38.824 | 27.246 |
| breastfeeding: formula | -9.770 | 14.701 | -0.665 | 0.510 | -39.379 | 19.839 |
| c_section: No | 2.659 | 7.823 | 0.340 | 0.736 | -13.097 | 18.414 |
| c_section: Yes | 4.588 | 10.097 | 0.454 | 0.652 | -15.748 | 24.924 |
| antibiotics: No | 2.526 | 10.442 | 0.242 | 0.810 | -18.505 | 23.557 |
| antibiotics: Yes | -20.185 | 28.048 | -0.720 | 0.475 | -76.675 | 36.306 |
| covid_impact | 1.623 | 0.868 | 1.870 | 0.068 | -0.125 | 3.371 |
| log_cortisol | -0.272 | 3.449 | -0.079 | 0.938 | -7.218 | 6.674 |
| time_since_stable_care | -1.132 | 0.955 | -1.185 | 0.242 | -3.055 | 0.791 |

**Supplementary Table S2.3**

Association between time since entry into stable care and Bray-Curtis dissimilarity.

|  | **Df** | **SumOfSqs** | **R2** | **F** | **Pr(>F)** |
| --- | --- | --- | --- | --- | --- |
| child_age_years | 1 | 0.231 | 0.026 | 1.525 | 0.100 |
| child_sex | 1 | 0.138 | 0.016 | 0.911 | 0.495 |
| breastfeeding | 3 | 0.481 | 0.055 | 1.059 | 0.322 |
| c_section | 2 | 0.254 | 0.029 | 0.838 | 0.701 |
| antibiotics | 2 | 0.231 | 0.026 | 0.761 | 0.817 |
| covid_impact | 1 | 0.214 | 0.024 | 1.410 | 0.131 |
| time_since_stable_care | 1 | 0.255 | 0.029 | 1.683 | 0.070 |
| log_cortisol | 1 | 0.157 | 0.018 | 1.035 | 0.361 |
| Residual | 45 | 6.818 | 0.777 | NA | NA |
| Total | 57 | 8.779 | 1.000 | NA | NA |

**Supplementary Table S2.4**

Association between time since entry into stable care and Jaccard dissimilarity.

|  | **Df** | **SumOfSqs** | **R2** | **F** | **Pr(>F)** |
| --- | --- | --- | --- | --- | --- |
| child_age_years | 1 | 0.311 | 0.026 | 1.524 | 0.008 |
| child_sex | 1 | 0.192 | 0.016 | 0.938 | 0.618 |
| breastfeeding | 3 | 0.668 | 0.056 | 1.090 | 0.193 |
| c_section | 2 | 0.411 | 0.035 | 1.005 | 0.437 |
| antibiotics | 2 | 0.352 | 0.030 | 0.862 | 0.852 |
| covid_impact | 1 | 0.196 | 0.017 | 0.960 | 0.529 |
| time_since_stable_care | 1 | 0.322 | 0.027 | 1.575 | 0.008 |
| log_cortisol | 1 | 0.223 | 0.019 | 1.091 | 0.248 |
| Residual | 45 | 9.190 | 0.775 | NA | NA |
| Total | 57 | 11.864 | 1.000 | NA | NA |

**Supplementary Table S2.5**

Association between time since entry into stable care and unweighted Unifrac dissimilarity.

|  | **Df** | **SumOfSqs** | **R2** | **F** | **Pr(>F)** |
| --- | --- | --- | --- | --- | --- |
| child_age_years | 1 | 0.098 | 0.027 | 1.582 | 0.066 |
| child_sex | 1 | 0.067 | 0.019 | 1.083 | 0.321 |
| breastfeeding | 3 | 0.162 | 0.045 | 0.867 | 0.656 |
| c_section | 2 | 0.109 | 0.030 | 0.879 | 0.630 |
| antibiotics | 2 | 0.107 | 0.030 | 0.856 | 0.638 |
| covid_impact | 1 | 0.078 | 0.022 | 1.246 | 0.205 |
| time_since_stable_care | 1 | 0.094 | 0.026 | 1.518 | 0.101 |
| log_cortisol | 1 | 0.072 | 0.020 | 1.165 | 0.276 |
| Residual | 45 | 2.801 | 0.780 | NA | NA |
| Total | 57 | 3.589 | 1.000 | NA | NA |

**Supplementary Table S2.6**

Association time since entry into stable care and weighted Unifrac dissimilarity.

|  | **Df** | **SumOfSqs** | **R2** | **F** | **Pr(>F)** |
| --- | --- | --- | --- | --- | --- |
| child_age_years | 1 | 0.029 | 0.025 | 1.415 | 0.228 |
| child_sex | 1 | 0.009 | 0.007 | 0.413 | 0.816 |
| breastfeeding | 3 | 0.076 | 0.065 | 1.227 | 0.242 |
| c_section | 2 | 0.041 | 0.035 | 0.985 | 0.397 |
| antibiotics | 2 | 0.018 | 0.016 | 0.446 | 0.887 |
| covid_impact | 1 | 0.028 | 0.024 | 1.373 | 0.232 |
| time_since_stable_care | 1 | 0.024 | 0.020 | 1.149 | 0.306 |
| log_cortisol | 1 | 0.019 | 0.016 | 0.912 | 0.432 |
| Residual | 45 | 0.931 | 0.792 | NA | NA |
| Total | 57 | 1.176 | 1.000 | NA | NA |

**Supplement 3 – Coronavirus impact scale**

How concerned do you feel about COVID-19?

- Not at all concerned (1)
- A little concerned (2)
- Moderately concerned (3)
- Very concerned (4)
- Extremely concerned (5)

Have you made any changes to your daily lifestyle to reduce risk due to COVID-19?

- Yes (1)
- No (0)

How often are you getting outside of your house for allowed shelter-in-place activities (e.g., going on a walk or a run, walking a pet, spending time in your backyard, playing outdoor no-contact sports with members of your household)?

- Multiple times a day (1)
- Once a day (2)
- Every couple of days (3)
- Once a week (4)
- Less than once a week (5)

Have you been unable to work due to COVID-19 related work changes?

- Yes, completely (e.g., lost job or unable to run your business) (2)
- Yes, partially (e.g., not working, but still being paid or able to do some work, but reduced hours) (1)
- No (e.g., still able to work same amount or more, even if now working remotely) (0)
- NA

Have you lost income due to COVID-19 related work changes?

- Yes, completely (2)
- Yes, partially (1)
- No (0)

*The scale is the sum of these items, with the value for each response given in parentheses, ranging from 2-15. Participants who enrolled prior to the coronavirus-2019 pandemic or who did not complete the scale were given a 0, such that the final range of the scale is 0-15.*

**Supplement 4 – Caregiver education and hair cortisol**

**Table S4.1**

Effect of highest caregiver education on children’s hair cortisol

| **term** | **estimate** | **std_error** | **statistic** | **p_value** | **lower_ci** | **upper_ci** |
| --- | --- | --- | --- | --- | --- | --- |
| intercept | 1.972 | 0.193 | 10.195 | 0.000 | 1.589 | 2.355 |
| Bachelors degree > no bachelors | -0.151 | 0.250 | -0.606 | 0.545 | -0.645 | 0.343 |
| Graduate degree > bachelors | 0.149 | 0.193 | 0.770 | 0.443 | -0.233 | 0.530 |

*Note:* highest caregiver education refers to the highest education (graduate degree, bachelor’s degree, or less than bachelor’s) attained by either of the child’s caregivers.

Education was sequentially coded for this model, with the reference category being less than a bachelor’s degree.

**Supplement 5 – Oral hygiene and oral health scale**

**Oral Health Questionnaire**

1. Do you/does your child have any of the following problems with your/their teeth or mouth?

*(Check all that apply; mark 🗸 on each line; skip if none apply)*

| Bleeding gums |  |
| --- | --- |
| Decayed tooth/teeth (dental caries or cavities, including filled cavities) |  |
| Tooth ache |  |
| Your/their gums are sore when brushing |  |

2. How often do you/does your child do the following?

| ***Frequency*** | ***Brushing*** | ***Flossing*** |
| --- | --- | --- |
| 2 or more times a day |  |  |
| Once a day |  |  |
| A few times a week |  |  |
| About once a week |  |  |
| Less than once a week |  |  |
| Never |  |  |

(Scale for each item ranges from never = 0 to 2 or more times a day = 5; brushing and flossing are summed to create the oral hygiene score)

Adapted from Simpson et al., 2020.

C. A. Simpson, *et al.*, Oral microbiome composition, but not diversity, is associated with adolescent anxiety and depression symptoms. *Physiology & Behavior* **226**, 113126 (2020).

**Supplement 6 – Effects of cortisol controlling for oral hygiene behaviors**

**Supplementary Table S6.1**

Association between the interaction between cortisol and CA group and Faith’s Phylogenetic Diversity, adjusting for oral hygiene.

| **term** | **estimate** | **std_error** | **statistic** | **p_value** | **lower_ci** | **upper_ci** |
| --- | --- | --- | --- | --- | --- | --- |
| intercept | 12.003 | 2.168 | 5.536 | 0.000 | 7.650 | 16.355 |
| child_age_years | 0.080 | 0.100 | 0.804 | 0.425 | -0.120 | 0.280 |
| child_sex: male | 0.572 | 0.563 | 1.016 | 0.315 | -0.559 | 1.703 |
| breastfeeding: combination | 0.082 | 0.881 | 0.094 | 0.926 | -1.686 | 1.851 |
| breastfeeding: DK or NA | 1.283 | 1.461 | 0.878 | 0.384 | -1.650 | 4.217 |
| breastfeeding: formula | 1.790 | 1.194 | 1.499 | 0.140 | -0.607 | 4.186 |
| c_section: No | -0.252 | 0.956 | -0.264 | 0.793 | -2.171 | 1.667 |
| c_section: Yes | -0.280 | 1.073 | -0.261 | 0.795 | -2.433 | 1.874 |
| antibiotics: No | 0.543 | 0.952 | 0.570 | 0.571 | -1.369 | 2.455 |
| antibiotics: Yes | -0.230 | 1.363 | -0.169 | 0.867 | -2.966 | 2.506 |
| covid_impact | -0.014 | 0.081 | -0.169 | 0.867 | -0.176 | 0.149 |
| Adversity: Caregiving Adversity | -4.123 | 1.693 | -2.435 | 0.018 | -7.523 | -0.724 |
| log_cortisol | -0.797 | 0.398 | -2.004 | 0.050 | -1.595 | 0.001 |
| oral_hygiene | -0.039 | 0.160 | -0.242 | 0.810 | -0.360 | 0.283 |
| Adversity: Caregiving Adversity:log_cortisol | 1.325 | 0.632 | 2.098 | 0.041 | 0.057 | 2.593 |

**Supplementary Table S6.2**

Association between the interaction between cortisol and CA group and observed feature counts, adjusting for oral hygiene.

| **term** | **estimate** | **std_error** | **statistic** | **p_value** | **lower_ci** | **upper_ci** |
| --- | --- | --- | --- | --- | --- | --- |
| intercept | 85.511 | 26.473 | 3.230 | 0.002 | 32.365 | 138.658 |
| child_age_years | 2.313 | 1.217 | 1.900 | 0.063 | -0.130 | 4.756 |
| child_sex: male | 5.761 | 6.879 | 0.837 | 0.406 | -8.049 | 19.571 |
| breastfeeding: combination | -14.700 | 10.758 | -1.366 | 0.178 | -36.298 | 6.898 |
| breastfeeding: DK or NA | 24.249 | 17.841 | 1.359 | 0.180 | -11.568 | 60.066 |
| breastfeeding: formula | 17.729 | 14.575 | 1.216 | 0.229 | -11.532 | 46.989 |
| c_section: No | 1.423 | 11.673 | 0.122 | 0.903 | -22.013 | 24.859 |
| c_section: Yes | -2.556 | 13.098 | -0.195 | 0.846 | -28.851 | 23.738 |
| antibiotics: No | 12.114 | 11.628 | 1.042 | 0.302 | -11.230 | 35.458 |
| antibiotics: Yes | 7.503 | 16.639 | 0.451 | 0.654 | -25.902 | 40.907 |
| covid_impact | 0.102 | 0.988 | 0.103 | 0.918 | -1.881 | 2.085 |
| Adversity: Caregiving Adversity | -31.790 | 20.678 | -1.537 | 0.130 | -73.303 | 9.722 |
| log_cortisol | -5.409 | 4.855 | -1.114 | 0.270 | -15.156 | 4.338 |
| oral_hygiene | -0.219 | 1.955 | -0.112 | 0.911 | -4.144 | 3.706 |
| Adversity: Caregiving Adversity:log_cortisol | 7.780 | 7.712 | 1.009 | 0.318 | -7.703 | 23.263 |

|  |  |  |  |  |  |  |
| --- | --- | --- | --- | --- | --- | --- |

**Supplementary Table S6.3**

Association between the interaction between cortisol and CA group and Jaccard dissimilarity.

|  | **Df** | **SumOfSqs** | **R2** | **F** | **Pr(>F)** |
| --- | --- | --- | --- | --- | --- |
| child_age_years | 1 | 0.329 | 0.024 | 1.572 | 0.008 |
| child_sex | 1 | 0.173 | 0.013 | 0.828 | 0.874 |
| breastfeeding | 3 | 0.695 | 0.050 | 1.106 | 0.166 |
| c_section | 2 | 0.417 | 0.030 | 0.994 | 0.475 |
| antibiotics | 2 | 0.435 | 0.031 | 1.038 | 0.360 |
| covid_impact | 1 | 0.213 | 0.015 | 1.015 | 0.387 |
| oral_hygeine | 1 | 0.206 | 0.015 | 0.983 | 0.476 |
| Adversity | 1 | 0.221 | 0.016 | 1.053 | 0.353 |
| log_cortisol | 1 | 0.269 | 0.019 | 1.283 | 0.073 |
| Adversity:log_cortisol | 1 | 0.188 | 0.014 | 0.899 | 0.693 |
| Residual | 51 | 10.687 | 0.773 | NA | NA |
| Total | 65 | 13.833 | 1.000 | NA | NA |

**Supplementary Table S6.4**

Association between the interaction between cortisol and CA group and unweighted Unifrac dissimilarity.

|  | **Df** | **SumOfSqs** | **R2** | **F** | **Pr(>F)** |
| --- | --- | --- | --- | --- | --- |
| child_age_years | 1 | 0.101 | 0.023 | 1.471 | 0.095 |
| child_sex | 1 | 0.060 | 0.014 | 0.877 | 0.561 |
| breastfeeding | 3 | 0.209 | 0.047 | 1.014 | 0.436 |
| c_section | 2 | 0.111 | 0.025 | 0.807 | 0.742 |
| antibiotics | 2 | 0.112 | 0.026 | 0.818 | 0.737 |
| covid_impact | 1 | 0.042 | 0.009 | 0.609 | 0.911 |
| oral_hygeine | 1 | 0.043 | 0.010 | 0.622 | 0.889 |
| Adversity | 1 | 0.075 | 0.017 | 1.088 | 0.322 |
| log_cortisol | 1 | 0.065 | 0.015 | 0.950 | 0.499 |
| Adversity:log_cortisol | 1 | 0.085 | 0.019 | 1.243 | 0.212 |
| Residual | 51 | 3.498 | 0.795 | NA | NA |
| Total | 65 | 4.401 | 1.000 | NA | NA |

**Supplement 7 – Effect of in-person vs. remote participation**

**Supplementary Table S7.1**

Association between the interaction between cortisol and CA group and Faith’s Phylogenetic Diversity, adjusting for online/in-person participation.

| **term** | **estimate** | **std_error** | **statistic** | **p_value** | **lower_ci** | **upper_ci** |
| --- | --- | --- | --- | --- | --- | --- |
| intercept | 10.553 | 0.931 | 11.331 | 0.000 | 8.710 | 12.396 |
| child_age_years | 0.069 | 0.048 | 1.427 | 0.156 | -0.027 | 0.164 |
| child_sex: male | 0.247 | 0.326 | 0.758 | 0.450 | -0.397 | 0.891 |
| breastfeeding: combination | 0.109 | 0.478 | 0.228 | 0.820 | -0.837 | 1.055 |
| breastfeeding: DK or NA | 0.692 | 0.807 | 0.857 | 0.393 | -0.905 | 2.289 |
| breastfeeding: formula | 0.762 | 0.615 | 1.238 | 0.218 | -0.456 | 1.980 |
| c_section: No | -0.665 | 0.558 | -1.192 | 0.236 | -1.769 | 0.439 |
| c_section: Yes | -0.396 | 0.595 | -0.666 | 0.507 | -1.574 | 0.781 |
| antibiotics: No | 0.557 | 0.493 | 1.130 | 0.261 | -0.419 | 1.534 |
| antibiotics: Yes | 0.469 | 0.766 | 0.612 | 0.542 | -1.047 | 1.985 |
| covid_impact | -0.049 | 0.053 | -0.915 | 0.362 | -0.154 | 0.057 |
| Adversity: Caregiving Adversity | -1.557 | 0.908 | -1.716 | 0.089 | -3.354 | 0.239 |
| log_cortisol | -0.676 | 0.222 | -3.045 | 0.003 | -1.115 | -0.237 |
| wave_1_online | 1.350 | 0.542 | 2.493 | 0.014 | 0.278 | 2.422 |
| Adversity: Caregiving Adversity:log_cortisol | 0.697 | 0.363 | 1.919 | 0.057 | -0.022 | 1.416 |

**Supplementary Table S7.2**

Association between the interaction between cortisol and CA group and observed feature counts, adjusting for online/in-person participation.

| **term** | **estimate** | **std_error** | **statistic** | **p_value** | **lower_ci** | **upper_ci** |
| --- | --- | --- | --- | --- | --- | --- |
| intercept | 84.682 | 11.059 | 7.657 | 0.000 | 62.796 | 106.567 |
| child_age_years | 1.351 | 0.572 | 2.362 | 0.020 | 0.219 | 2.482 |
| child_sex: male | 2.349 | 3.865 | 0.608 | 0.544 | -5.300 | 9.998 |
| breastfeeding: combination | -6.197 | 5.675 | -1.092 | 0.277 | -17.428 | 5.034 |
| breastfeeding: DK or NA | 15.293 | 9.580 | 1.596 | 0.113 | -3.666 | 34.253 |
| breastfeeding: formula | 10.553 | 7.307 | 1.444 | 0.151 | -3.908 | 25.013 |
| c_section: No | 0.691 | 6.625 | 0.104 | 0.917 | -12.421 | 13.803 |
| c_section: Yes | 1.719 | 7.064 | 0.243 | 0.808 | -12.260 | 15.698 |
| antibiotics: No | 7.575 | 5.859 | 1.293 | 0.198 | -4.020 | 19.170 |
| antibiotics: Yes | 11.713 | 9.095 | 1.288 | 0.200 | -6.285 | 29.711 |
| covid_impact | -0.505 | 0.632 | -0.799 | 0.426 | -1.757 | 0.746 |
| Adversity: Caregiving Adversity | -15.722 | 10.777 | -1.459 | 0.147 | -37.050 | 5.606 |
| log_cortisol | -6.687 | 2.636 | -2.536 | 0.012 | -11.904 | -1.469 |
| wave_1_online | 15.581 | 6.430 | 2.423 | 0.017 | 2.856 | 28.305 |
| Adversity: Caregiving Adversity:log_cortisol | 6.133 | 4.314 | 1.422 | 0.158 | -2.404 | 14.671 |

**Supplementary Table S7.3**

Association between the interaction between cortisol and CA group and Bray-Curtis dissimilarity.

|  | **Df** | **SumOfSqs** | **R2** | **F** | **Pr(>F)** |
| --- | --- | --- | --- | --- | --- |
| child_age_years | 1 | 0.270 | 0.013 | 1.813 | 0.043 |
| child_sex | 1 | 0.142 | 0.007 | 0.951 | 0.461 |
| breastfeeding | 3 | 0.508 | 0.024 | 1.137 | 0.243 |
| c_section | 2 | 0.363 | 0.017 | 1.219 | 0.185 |
| antibiotics | 2 | 0.417 | 0.019 | 1.401 | 0.083 |
| covid_impact | 1 | 0.165 | 0.008 | 1.106 | 0.322 |
| wave_1_online | 1 | 0.216 | 0.010 | 1.448 | 0.104 |
| Adversity | 1 | 0.306 | 0.014 | 2.056 | 0.016 |
| log_cortisol | 1 | 0.148 | 0.007 | 0.992 | 0.412 |
| Adversity:log_cortisol | 1 | 0.176 | 0.008 | 1.179 | 0.260 |
| Residual | 126 | 18.773 | 0.874 | NA | NA |
| Total | 140 | 21.484 | 1.000 | NA | NA |

**Supplementary Table S7.4**

Association between the interaction between cortisol and CA group and Jaccard dissimilarity.

|  | **Df** | **SumOfSqs** | **R2** | **F** | **Pr(>F)** |
| --- | --- | --- | --- | --- | --- |
| child_age_years | 1 | 0.450 | 0.016 | 2.25 | 0.001 |
| child_sex | 1 | 0.194 | 0.007 | 0.97 | 0.530 |
| breastfeeding | 3 | 0.748 | 0.026 | 1.25 | 0.017 |
| c_section | 2 | 0.483 | 0.017 | 1.21 | 0.049 |
| antibiotics | 2 | 0.441 | 0.015 | 1.10 | 0.176 |
| covid_impact | 1 | 0.216 | 0.007 | 1.08 | 0.251 |
| wave_1_online | 1 | 0.320 | 0.011 | 1.60 | 0.005 |
| Adversity | 1 | 0.292 | 0.010 | 1.46 | 0.029 |
| log_cortisol | 1 | 0.273 | 0.009 | 1.36 | 0.040 |
| Adversity:log_cortisol | 1 | 0.239 | 0.008 | 1.19 | 0.122 |
| Residual | 126 | 25.239 | 0.873 | NA | NA |
| Total | 140 | 28.895 | 1.000 | NA | NA |

**Supplementary Table S7.5**

Association between the interaction between cortisol and CA group and unweighted Unifrac dissimilarity.

|  | **Df** | **SumOfSqs** | **R2** | **F** | **Pr(>F)** |
| --- | --- | --- | --- | --- | --- |
| child_age_years | 1 | 0.141 | 0.017 | 2.432 | 0.003 |
| child_sex | 1 | 0.051 | 0.006 | 0.881 | 0.537 |
| breastfeeding | 3 | 0.197 | 0.024 | 1.132 | 0.232 |
| c_section | 2 | 0.101 | 0.012 | 0.871 | 0.669 |
| antibiotics | 2 | 0.128 | 0.015 | 1.099 | 0.312 |
| covid_impact | 1 | 0.040 | 0.005 | 0.693 | 0.819 |
| wave_1_online | 1 | 0.096 | 0.012 | 1.648 | 0.057 |
| Adversity | 1 | 0.074 | 0.009 | 1.282 | 0.191 |
| log_cortisol | 1 | 0.078 | 0.009 | 1.341 | 0.158 |
| Adversity:log_cortisol | 1 | 0.084 | 0.010 | 1.447 | 0.109 |
| Residual | 126 | 7.314 | 0.881 | NA | NA |
| Total | 140 | 8.304 | 1.000 | NA | NA |

**Supplementary Table S7.6**

Association between the interaction between cortisol and CA group and weighted Unifrac dissimilarity.

|  | **Df** | **SumOfSqs** | **R2** | **F** | **Pr(>F)** |
| --- | --- | --- | --- | --- | --- |
| child_age_years | 1 | 0.039 | 0.014 | 2.033 | 0.067 |
| child_sex | 1 | 0.013 | 0.005 | 0.673 | 0.626 |
| breastfeeding | 3 | 0.045 | 0.016 | 0.780 | 0.661 |
| c_section | 2 | 0.072 | 0.026 | 1.854 | 0.062 |
| antibiotics | 2 | 0.049 | 0.017 | 1.260 | 0.260 |
| covid_impact | 1 | 0.030 | 0.011 | 1.550 | 0.178 |
| wave_1_online | 1 | 0.021 | 0.008 | 1.097 | 0.330 |
| Adversity | 1 | 0.045 | 0.016 | 2.320 | 0.049 |
| log_cortisol | 1 | 0.043 | 0.015 | 2.234 | 0.070 |
| Adversity:log_cortisol | 1 | 0.017 | 0.006 | 0.856 | 0.492 |
| Residual | 126 | 2.433 | 0.867 | NA | NA |
| Total | 140 | 2.806 | 1.000 | NA | NA |

**Supplement 8 – Hair storage temperature**

**Supplementary Table S8.1**

Association between the interaction between cortisol and CA group and Faith’s Phylogenetic Diversity, adjusting for hair sample storage temperature.

| **term** | **estimate** | **std_error** | **statistic** | **p_value** | **lower_ci** | **upper_ci** |
| --- | --- | --- | --- | --- | --- | --- |
| intercept | 9.636 | 1.270 | 7.589 | 0.000 | 7.123 | 12.148 |
| child_age_years | 0.075 | 0.049 | 1.541 | 0.126 | -0.021 | 0.172 |
| child_sex: male | 0.320 | 0.330 | 0.967 | 0.335 | -0.334 | 0.973 |
| breastfeeding: combination | 0.129 | 0.487 | 0.266 | 0.791 | -0.834 | 1.093 |
| breastfeeding: DK or NA | 0.943 | 0.813 | 1.161 | 0.248 | -0.665 | 2.552 |
| breastfeeding: formula | 0.971 | 0.619 | 1.570 | 0.119 | -0.253 | 2.195 |
| c_section: No | -0.540 | 0.565 | -0.955 | 0.341 | -1.659 | 0.579 |
| c_section: Yes | -0.196 | 0.598 | -0.327 | 0.744 | -1.379 | 0.988 |
| antibiotics: No | 0.650 | 0.501 | 1.299 | 0.196 | -0.341 | 1.641 |
| antibiotics: Yes | 0.621 | 0.776 | 0.799 | 0.426 | -0.916 | 2.157 |
| covid_impact | 0.018 | 0.045 | 0.406 | 0.685 | -0.070 | 0.106 |
| Adversity: Caregiving Adversity | -1.556 | 0.930 | -1.673 | 0.097 | -3.397 | 0.285 |
| log_cortisol | -0.641 | 0.226 | -2.837 | 0.005 | -1.088 | -0.194 |
| hair_storage_temperature: room temperature | 1.256 | 0.997 | 1.261 | 0.210 | -0.716 | 3.229 |
| Adversity: Caregiving Adversity:log_cortisol | 0.739 | 0.372 | 1.985 | 0.049 | 0.002 | 1.475 |

**Supplementary Table S8.2**

Association between the interaction between cortisol and CA group and observed feature counts, adjusting for hair sample storage temperature.

| **term** | **estimate** | **std_error** | **statistic** | **p_value** | **lower_ci** | **upper_ci** |
| --- | --- | --- | --- | --- | --- | --- |
| intercept | 70.878 | 15.008 | 4.723 | 0.000 | 41.177 | 100.578 |
| child_age_years | 1.423 | 0.578 | 2.462 | 0.015 | 0.279 | 2.568 |
| child_sex: male | 3.210 | 3.905 | 0.822 | 0.413 | -4.517 | 10.937 |
| breastfeeding: combination | -6.052 | 5.753 | -1.052 | 0.295 | -17.438 | 5.333 |
| breastfeeding: DK or NA | 18.015 | 9.608 | 1.875 | 0.063 | -0.999 | 37.028 |
| breastfeeding: formula | 12.770 | 7.311 | 1.747 | 0.083 | -1.699 | 27.239 |
| c_section: No | 1.892 | 6.683 | 0.283 | 0.778 | -11.334 | 15.117 |
| c_section: Yes | 3.833 | 7.069 | 0.542 | 0.589 | -10.157 | 17.822 |
| antibiotics: No | 8.611 | 5.917 | 1.455 | 0.148 | -3.100 | 20.321 |
| antibiotics: Yes | 13.333 | 9.177 | 1.453 | 0.149 | -4.828 | 31.495 |
| covid_impact | 0.227 | 0.526 | 0.431 | 0.667 | -0.815 | 1.268 |
| Adversity: Caregiving Adversity | -15.229 | 10.997 | -1.385 | 0.169 | -36.992 | 6.533 |
| log_cortisol | -6.250 | 2.671 | -2.340 | 0.021 | -11.537 | -0.964 |
| hair_storage_temperature: room temperature | 18.283 | 11.780 | 1.552 | 0.123 | -5.030 | 41.595 |
| Adversity: Caregiving Adversity:log_cortisol | 6.365 | 4.399 | 1.447 | 0.150 | -2.341 | 15.071 |

**Supplementary Table S8.3**

Association between the interaction between cortisol and CA group and Jaccard dissimilarity.

|  | **Df** | **SumOfSqs** | **R2** | **F** | **Pr(>F)** |
| --- | --- | --- | --- | --- | --- |
| child_age_years | 1 | 0.450 | 0.016 | 2.237 | 0.001 |
| child_sex | 1 | 0.194 | 0.007 | 0.966 | 0.536 |
| breastfeeding | 3 | 0.748 | 0.026 | 1.240 | 0.025 |
| c_section | 2 | 0.483 | 0.017 | 1.202 | 0.057 |
| antibiotics | 2 | 0.441 | 0.015 | 1.096 | 0.209 |
| covid_impact | 1 | 0.216 | 0.007 | 1.073 | 0.286 |
| hair_storage_temperature | 1 | 0.235 | 0.008 | 1.170 | 0.180 |
| Adversity | 1 | 0.295 | 0.010 | 1.468 | 0.013 |
| log_cortisol | 1 | 0.251 | 0.009 | 1.248 | 0.078 |
| Adversity:log_cortisol | 1 | 0.251 | 0.009 | 1.248 | 0.080 |
| Residual | 126 | 25.332 | 0.877 | NA | NA |
| Total | 140 | 28.895 | 1.000 | NA | NA |

**Supplementary Table S8.4**

Association between the interaction between cortisol and CA group and unweighted Unifrac dissimilarity.

|  | **Df** | **SumOfSqs** | **R2** | **F** | **Pr(>F)** |
| --- | --- | --- | --- | --- | --- |
| child_age_years | 1 | 0.141 | 0.017 | 2.422 | 0.007 |
| child_sex | 1 | 0.051 | 0.006 | 0.877 | 0.593 |
| breastfeeding | 3 | 0.197 | 0.024 | 1.127 | 0.253 |
| c_section | 2 | 0.101 | 0.012 | 0.868 | 0.669 |
| antibiotics | 2 | 0.128 | 0.015 | 1.094 | 0.304 |
| covid_impact | 1 | 0.040 | 0.005 | 0.690 | 0.829 |
| hair_storage_temperature | 1 | 0.069 | 0.008 | 1.188 | 0.221 |
| Adversity | 1 | 0.076 | 0.009 | 1.297 | 0.188 |
| log_cortisol | 1 | 0.068 | 0.008 | 1.170 | 0.261 |
| Adversity:log_cortisol | 1 | 0.089 | 0.011 | 1.524 | 0.102 |
| Residual | 126 | 7.344 | 0.884 | NA | NA |
| Total | 140 | 8.304 | 1.000 | NA | NA |

**Supplement 9 – Saliva storage/incubation protocol variations**

**Supplementary Table S9.1**

Association between the interaction between cortisol and CA group and Faith’s Phylogenetic Diversity. The first table shows adjustment for saliva storage time; the second shows adjustment for whether the sample was incubated.

| **term** | **estimate** | **std_error** | **statistic** | **p_value** | **lower_ci** | **upper_ci** |
| --- | --- | --- | --- | --- | --- | --- |
| intercept | 11.284 | 1.045 | 10.800 | 0.000 | 9.217 | 13.352 |
| child_age_years | 0.074 | 0.049 | 1.503 | 0.135 | -0.023 | 0.170 |
| child_sex: male | 0.299 | 0.330 | 0.906 | 0.367 | -0.354 | 0.953 |
| breastfeeding: combination | 0.145 | 0.486 | 0.299 | 0.766 | -0.817 | 1.107 |
| breastfeeding: DK or NA | 0.974 | 0.811 | 1.200 | 0.232 | -0.632 | 2.579 |
| breastfeeding: formula | 0.969 | 0.618 | 1.568 | 0.119 | -0.254 | 2.193 |
| c_section: No | -0.498 | 0.562 | -0.885 | 0.378 | -1.610 | 0.615 |
| c_section: Yes | -0.182 | 0.597 | -0.304 | 0.761 | -1.363 | 0.999 |
| antibiotics: No | 0.665 | 0.500 | 1.330 | 0.186 | -0.325 | 1.656 |
| antibiotics: Yes | 0.615 | 0.776 | 0.792 | 0.430 | -0.921 | 2.151 |
| covid_impact | 0.004 | 0.048 | 0.092 | 0.927 | -0.090 | 0.099 |
| Adversity: Caregiving Adversity | -1.729 | 0.921 | -1.877 | 0.063 | -3.553 | 0.094 |
| log_cortisol | -0.675 | 0.226 | -2.982 | 0.003 | -1.123 | -0.227 |
| saliva_storage_time | -0.002 | 0.001 | -1.308 | 0.193 | -0.005 | 0.001 |
| Adversity: Caregiving Adversity:log_cortisol | 0.809 | 0.366 | 2.208 | 0.029 | 0.084 | 1.534 |

| **term** | **estimate** | **std_error** | **statistic** | **p_value** | **lower_ci** | **upper_ci** |
| --- | --- | --- | --- | --- | --- | --- |
| intercept | 9.636 | 1.270 | 7.589 | 0.000 | 7.123 | 12.148 |
| child_age_years | 0.075 | 0.049 | 1.541 | 0.126 | -0.021 | 0.172 |
| child_sex: male | 0.320 | 0.330 | 0.967 | 0.335 | -0.334 | 0.973 |
| breastfeeding: combination | 0.129 | 0.487 | 0.266 | 0.791 | -0.834 | 1.093 |
| breastfeeding: DK or NA | 0.943 | 0.813 | 1.161 | 0.248 | -0.665 | 2.552 |
| breastfeeding: formula | 0.971 | 0.619 | 1.570 | 0.119 | -0.253 | 2.195 |
| c_section: No | -0.540 | 0.565 | -0.955 | 0.341 | -1.659 | 0.579 |
| c_section: Yes | -0.196 | 0.598 | -0.327 | 0.744 | -1.379 | 0.988 |
| antibiotics: No | 0.650 | 0.501 | 1.299 | 0.196 | -0.341 | 1.641 |
| antibiotics: Yes | 0.621 | 0.776 | 0.799 | 0.426 | -0.916 | 2.157 |
| covid_impact | 0.018 | 0.045 | 0.406 | 0.685 | -0.070 | 0.106 |
| Adversity: Caregiving Adversity | -1.556 | 0.930 | -1.673 | 0.097 | -3.397 | 0.285 |
| log_cortisol | -0.641 | 0.226 | -2.837 | 0.005 | -1.088 | -0.194 |
| saliva_incubated | 1.256 | 0.997 | 1.261 | 0.210 | -0.716 | 3.229 |
| Adversity: Caregiving Adversity:log_cortisol | 0.739 | 0.372 | 1.985 | 0.049 | 0.002 | 1.475 |

**Supplementary Table S9.2**

Association between the interaction between cortisol and CA group and observed feature counts. The first table shows adjustment for saliva storage time; the second shows adjustment for whether the sample was incubated.

| **term** | **estimate** | **std_error** | **statistic** | **p_value** | **lower_ci** | **upper_ci** |
| --- | --- | --- | --- | --- | --- | --- |
| intercept | 89.848 | 12.453 | 7.215 | 0.000 | 65.203 | 114.492 |
| child_age_years | 1.425 | 0.583 | 2.444 | 0.016 | 0.271 | 2.580 |
| child_sex: male | 3.031 | 3.937 | 0.770 | 0.443 | -4.760 | 10.821 |
| breastfeeding: combination | -5.691 | 5.792 | -0.982 | 0.328 | -17.154 | 5.772 |
| breastfeeding: DK or NA | 18.720 | 9.670 | 1.936 | 0.055 | -0.417 | 37.856 |
| breastfeeding: formula | 13.317 | 7.368 | 1.807 | 0.073 | -1.265 | 27.898 |
| c_section: No | 2.832 | 6.702 | 0.423 | 0.673 | -10.431 | 16.094 |
| c_section: Yes | 4.498 | 7.112 | 0.632 | 0.528 | -9.577 | 18.574 |
| antibiotics: No | 8.800 | 5.963 | 1.476 | 0.142 | -3.000 | 20.600 |
| antibiotics: Yes | 13.679 | 9.251 | 1.479 | 0.142 | -4.628 | 31.986 |
| covid_impact | 0.258 | 0.570 | 0.452 | 0.652 | -0.871 | 1.387 |
| Adversity: Caregiving Adversity | -17.627 | 10.981 | -1.605 | 0.111 | -39.359 | 4.104 |
| log_cortisol | -6.547 | 2.698 | -2.427 | 0.017 | -11.886 | -1.207 |
| saliva_storage_time | -0.011 | 0.017 | -0.649 | 0.517 | -0.045 | 0.023 |
| Adversity: Caregiving Adversity:log_cortisol | 7.491 | 4.366 | 1.716 | 0.089 | -1.149 | 16.132 |

| **term** | **estimate** | **std_error** | **statistic** | **p_value** | **lower_ci** | **upper_ci** |
| --- | --- | --- | --- | --- | --- | --- |
| intercept | 70.878 | 15.008 | 4.723 | 0.000 | 41.177 | 100.578 |
| child_age_years | 1.423 | 0.578 | 2.462 | 0.015 | 0.279 | 2.568 |
| child_sex: male | 3.210 | 3.905 | 0.822 | 0.413 | -4.517 | 10.937 |
| breastfeeding: combination | -6.052 | 5.753 | -1.052 | 0.295 | -17.438 | 5.333 |
| breastfeeding: DK or NA | 18.015 | 9.608 | 1.875 | 0.063 | -0.999 | 37.028 |
| breastfeeding: formula | 12.770 | 7.311 | 1.747 | 0.083 | -1.699 | 27.239 |
| c_section: No | 1.892 | 6.683 | 0.283 | 0.778 | -11.334 | 15.117 |
| c_section: Yes | 3.833 | 7.069 | 0.542 | 0.589 | -10.157 | 17.822 |
| antibiotics: No | 8.611 | 5.917 | 1.455 | 0.148 | -3.100 | 20.321 |
| antibiotics: Yes | 13.333 | 9.177 | 1.453 | 0.149 | -4.828 | 31.495 |
| covid_impact | 0.227 | 0.526 | 0.431 | 0.667 | -0.815 | 1.268 |
| Adversity: Caregiving Adversity | -15.229 | 10.997 | -1.385 | 0.169 | -36.992 | 6.533 |
| log_cortisol | -6.250 | 2.671 | -2.340 | 0.021 | -11.537 | -0.964 |
| saliva_incubated | 18.283 | 11.780 | 1.552 | 0.123 | -5.030 | 41.595 |
| Adversity: Caregiving Adversity:log_cortisol | 6.365 | 4.399 | 1.447 | 0.150 | -2.341 | 15.071 |

**Supplementary Table S9.3**

Association between the interaction between cortisol and CA group and Jaccard dissimilarity. The first table shows adjustment for saliva storage time; the second shows adjustment for whether the sample was incubated.

|  | **Df** | **SumOfSqs** | **R2** | **F** | **Pr(>F)** |
| --- | --- | --- | --- | --- | --- |
| child_age_years | 1 | 0.450 | 0.016 | 2.234 | 0.001 |
| child_sex | 1 | 0.194 | 0.007 | 0.965 | 0.536 |
| breastfeeding | 3 | 0.748 | 0.026 | 1.238 | 0.026 |
| c_section | 2 | 0.483 | 0.017 | 1.200 | 0.058 |
| antibiotics | 2 | 0.441 | 0.015 | 1.094 | 0.213 |
| covid_impact | 1 | 0.216 | 0.007 | 1.071 | 0.290 |
| saliva_storage_time | 1 | 0.187 | 0.006 | 0.928 | 0.609 |
| Adversity | 1 | 0.293 | 0.010 | 1.458 | 0.014 |
| log_cort | 1 | 0.256 | 0.009 | 1.271 | 0.071 |
| Adversity:log_cort | 1 | 0.264 | 0.009 | 1.314 | 0.049 |
| Residual | 126 | 25.363 | 0.878 | NA | NA |
| Total | 140 | 28.895 | 1.000 | NA | NA |

|  | **Df** | **SumOfSqs** | **R2** | **F** | **Pr(>F)** |
| --- | --- | --- | --- | --- | --- |
| child_age_years | 1 | 0.450 | 0.016 | 2.237 | 0.001 |
| child_sex | 1 | 0.194 | 0.007 | 0.966 | 0.536 |
| breastfeeding | 3 | 0.748 | 0.026 | 1.240 | 0.025 |
| c_section | 2 | 0.483 | 0.017 | 1.202 | 0.057 |
| antibiotics | 2 | 0.441 | 0.015 | 1.096 | 0.209 |
| covid_impact | 1 | 0.216 | 0.007 | 1.073 | 0.286 |
| saliva_incubated | 1 | 0.235 | 0.008 | 1.170 | 0.180 |
| Adversity | 1 | 0.295 | 0.010 | 1.468 | 0.013 |
| log_cort | 1 | 0.251 | 0.009 | 1.248 | 0.078 |
| Adversity:log_cort | 1 | 0.251 | 0.009 | 1.248 | 0.080 |
| Residual | 126 | 25.332 | 0.877 | NA | NA |
| Total | 140 | 28.895 | 1.000 | NA | NA |

**Supplementary Table S9.4**

Association between the interaction between cortisol and CA group and unweighted Unifrac dissimilarity. The first table shows adjustment for saliva storage time; the second shows adjustment for whether the sample was incubated.

|  | **Df** | **SumOfSqs** | **R2** | **F** | **Pr(>F)** |
| --- | --- | --- | --- | --- | --- |
| child_age_years | 1 | 0.141 | 0.017 | 2.416 | 0.007 |
| child_sex | 1 | 0.051 | 0.006 | 0.875 | 0.594 |
| breastfeeding | 3 | 0.197 | 0.024 | 1.125 | 0.256 |
| c_section | 2 | 0.101 | 0.012 | 0.866 | 0.672 |
| antibiotics | 2 | 0.128 | 0.015 | 1.092 | 0.304 |
| covid_impact | 1 | 0.040 | 0.005 | 0.689 | 0.830 |
| saliva_storage_time | 1 | 0.045 | 0.005 | 0.767 | 0.732 |
| Adversity | 1 | 0.076 | 0.009 | 1.300 | 0.182 |
| log_cort | 1 | 0.069 | 0.008 | 1.174 | 0.258 |
| Adversity:log_cort | 1 | 0.097 | 0.012 | 1.653 | 0.068 |
| Residual | 126 | 7.360 | 0.886 | NA | NA |
| Total | 140 | 8.304 | 1.000 | NA | NA |

|  | **Df** | **SumOfSqs** | **R2** | **F** | **Pr(>F)** |
| --- | --- | --- | --- | --- | --- |
| child_age_years | 1 | 0.141 | 0.017 | 2.422 | 0.007 |
| child_sex | 1 | 0.051 | 0.006 | 0.877 | 0.593 |
| breastfeeding | 3 | 0.197 | 0.024 | 1.127 | 0.253 |
| c_section | 2 | 0.101 | 0.012 | 0.868 | 0.669 |
| antibiotics | 2 | 0.128 | 0.015 | 1.094 | 0.304 |
| covid_impact | 1 | 0.040 | 0.005 | 0.690 | 0.829 |
| saliva_incubated | 1 | 0.069 | 0.008 | 1.188 | 0.221 |
| Adversity | 1 | 0.076 | 0.009 | 1.297 | 0.188 |
| log_cort | 1 | 0.068 | 0.008 | 1.170 | 0.261 |
| Adversity:log_cort | 1 | 0.089 | 0.011 | 1.524 | 0.102 |
| Residual | 126 | 7.344 | 0.884 | NA | NA |
| Total | 140 | 8.304 | 1.000 | NA | NA |

**Supplementary Table S9.5**

Association between the interaction between cortisol and CA group and Bray-Curtis dissimilarity. The first table shows adjustment for saliva storage time; the second shows adjustment for whether the sample was incubated.

|  | **Df** | **SumOfSqs** | **R2** | **F** | **Pr(>F)** |
| --- | --- | --- | --- | --- | --- |
| child_age_years | 1 | 0.270 | 0.013 | 1.803 | 0.049 |
| child_sex | 1 | 0.142 | 0.007 | 0.946 | 0.493 |
| breastfeeding | 3 | 0.508 | 0.024 | 1.130 | 0.247 |
| c_section | 2 | 0.363 | 0.017 | 1.212 | 0.189 |
| antibiotics | 2 | 0.417 | 0.019 | 1.393 | 0.099 |
| covid_impact | 1 | 0.165 | 0.008 | 1.099 | 0.305 |
| saliva_storage_time | 1 | 0.112 | 0.005 | 0.751 | 0.750 |
| Adversity | 1 | 0.307 | 0.014 | 2.046 | 0.027 |
| log_cort | 1 | 0.138 | 0.006 | 0.918 | 0.509 |
| Adversity:log_cort | 1 | 0.180 | 0.008 | 1.202 | 0.235 |
| Residual | 126 | 18.882 | 0.879 | NA | NA |
| Total | 140 | 21.484 | 1.000 | NA | NA |

|  | **Df** | **SumOfSqs** | **R2** | **F** | **Pr(>F)** |
| --- | --- | --- | --- | --- | --- |
| child_age_years | 1 | 0.270 | 0.013 | 1.812 | 0.048 |
| child_sex | 1 | 0.142 | 0.007 | 0.951 | 0.488 |
| breastfeeding | 3 | 0.508 | 0.024 | 1.137 | 0.242 |
| c_section | 2 | 0.363 | 0.017 | 1.218 | 0.185 |
| antibiotics | 2 | 0.417 | 0.019 | 1.401 | 0.096 |
| covid_impact | 1 | 0.165 | 0.008 | 1.105 | 0.299 |
| saliva_incubated | 1 | 0.219 | 0.010 | 1.467 | 0.112 |
| Adversity | 1 | 0.308 | 0.014 | 2.065 | 0.026 |
| log_cort | 1 | 0.141 | 0.007 | 0.949 | 0.471 |
| Adversity:log_cort | 1 | 0.171 | 0.008 | 1.151 | 0.272 |
| Residual | 126 | 18.779 | 0.874 | NA | NA |
| Total | 140 | 21.484 | 1.000 | NA | NA |

**Supplementary Table S9.6**

Association between the interaction between cortisol and CA group and Weighted Unifrac dissimilarity. The first table shows adjustment for saliva storage time; the second shows adjustment for whether the sample was incubated.

|  | **Df** | **SumOfSqs** | **R2** | **F** | **Pr(>F)** |
| --- | --- | --- | --- | --- | --- |
| child_age_years | 1 | 0.039 | 0.014 | 2.025 | 0.069 |
| child_sex | 1 | 0.013 | 0.005 | 0.671 | 0.629 |
| breastfeeding | 3 | 0.045 | 0.016 | 0.777 | 0.666 |
| c_section | 2 | 0.072 | 0.026 | 1.847 | 0.064 |
| antibiotics | 2 | 0.049 | 0.017 | 1.255 | 0.261 |
| covid_impact | 1 | 0.030 | 0.011 | 1.544 | 0.180 |
| saliva_storage_time | 1 | 0.014 | 0.005 | 0.735 | 0.568 |
| Adversity | 1 | 0.045 | 0.016 | 2.329 | 0.045 |
| log_cort | 1 | 0.039 | 0.014 | 2.003 | 0.094 |
| Adversity:log_cort | 1 | 0.018 | 0.006 | 0.932 | 0.430 |
| Residual | 126 | 2.443 | 0.870 | NA | NA |
| Total | 140 | 2.806 | 1.000 | NA | NA |

|  | **Df** | **SumOfSqs** | **R2** | **F** | **Pr(>F)** |
| --- | --- | --- | --- | --- | --- |
| child_age_years | 1 | 0.039 | 0.014 | 2.026 | 0.068 |
| child_sex | 1 | 0.013 | 0.005 | 0.671 | 0.628 |
| breastfeeding | 3 | 0.045 | 0.016 | 0.777 | 0.666 |
| c_section | 2 | 0.072 | 0.026 | 1.848 | 0.063 |
| antibiotics | 2 | 0.049 | 0.017 | 1.256 | 0.262 |
| covid_impact | 1 | 0.030 | 0.011 | 1.545 | 0.178 |
| saliva_incubated | 1 | 0.017 | 0.006 | 0.889 | 0.422 |
| Adversity | 1 | 0.045 | 0.016 | 2.308 | 0.045 |
| log_cort | 1 | 0.040 | 0.014 | 2.064 | 0.091 |
| Adversity:log_cort | 1 | 0.016 | 0.006 | 0.833 | 0.500 |
| Residual | 126 | 2.441 | 0.870 | NA | NA |
| Total | 140 | 2.806 | 1.000 | NA | NA |

**Supplement 10 – Marginal method**

**Supplementary Table S10.1**

Association between the interaction between cortisol and CA group and beta diversity indices using the marginal method. The tables show, in order: Bray-Curtis, Jaccard, Unweighted Unifrac, and Weighted Unifrac indices.

|  | **Df** | **SumOfSqs** | **R2** | **F** | **Pr(>F)** |
| --- | --- | --- | --- | --- | --- |
| child_age_years | 1 | 0.2761950 | 0.0128559 | 1.8466941 | 0.041 |
| child_sex | 1 | 0.1122771 | 0.0052261 | 0.7507071 | 0.739 |
| breastfeeding | 3 | 0.4563149 | 0.0212398 | 1.0170038 | 0.426 |
| c_section | 2 | 0.4185600 | 0.0194824 | 1.3992873 | 0.076 |
| antibiotics | 2 | 0.4046770 | 0.0188362 | 1.3528753 | 0.101 |
| covid_impact | 1 | 0.1639750 | 0.0076324 | 1.0963692 | 0.331 |
| Adversity:log_cortisol | 1 | 0.1806395 | 0.0084081 | 1.2077916 | 0.255 |
| Residual | 127 | 18.9943532 | 0.8841171 | NA | NA |
| Total | 140 | 21.4839781 | 1.0000000 | NA | NA |

|  | **Df** | **SumOfSqs** | **R2** | **F** | **Pr(>F)** |
| --- | --- | --- | --- | --- | --- |
| child_age_years | 1 | 0.4458978 | 0.0154317 | 2.2158732 | 0.001 |
| child_sex | 1 | 0.1888753 | 0.0065366 | 0.9386089 | 0.620 |
| breastfeeding | 3 | 0.7142477 | 0.0247188 | 1.1831428 | 0.044 |
| c_section | 2 | 0.4972044 | 0.0172073 | 1.2354196 | 0.032 |
| antibiotics | 2 | 0.4473579 | 0.0154822 | 1.1115645 | 0.168 |
| covid_impact | 1 | 0.2246751 | 0.0077756 | 1.1165147 | 0.191 |
| Adversity:log_cortisol | 1 | 0.2677432 | 0.0092661 | 1.3305399 | 0.039 |
| Residual | 127 | 25.5560766 | 0.8844496 | NA | NA |
| Total | 140 | 28.8948943 | 1.0000000 | NA | NA |

|  | **Df** | **SumOfSqs** | **R2** | **F** | **Pr(>F)** |
| --- | --- | --- | --- | --- | --- |
| child_age_years | 1 | 0.1406318 | 0.0169351 | 2.4103913 | 0.002 |
| child_sex | 1 | 0.0520502 | 0.0062680 | 0.8921261 | 0.526 |
| breastfeeding | 3 | 0.2084316 | 0.0250997 | 1.1908200 | 0.188 |
| c_section | 2 | 0.1171273 | 0.0141047 | 1.0037651 | 0.456 |
| antibiotics | 2 | 0.1338950 | 0.0161239 | 1.1474616 | 0.254 |
| covid_impact | 1 | 0.0467788 | 0.0056332 | 0.8017765 | 0.671 |
| Adversity:log_cortisol | 1 | 0.0975332 | 0.0117451 | 1.6716932 | 0.055 |
| Residual | 127 | 7.4096864 | 0.8922867 | NA | NA |
| Total | 140 | 8.3041544 | 1.0000000 | NA | NA |

|  | **Df** | **SumOfSqs** | **R2** | **F** | **Pr(>F)** |
| --- | --- | --- | --- | --- | --- |
| child_age_years | 1 | 0.0358743 | 0.0127829 | 1.8530184 | 0.091 |
| child_sex | 1 | 0.0104656 | 0.0037292 | 0.5405822 | 0.728 |
| breastfeeding | 3 | 0.0606044 | 0.0215948 | 1.0434680 | 0.380 |
| c_section | 2 | 0.0758199 | 0.0270164 | 1.9581651 | 0.050 |
| antibiotics | 2 | 0.0448939 | 0.0159968 | 1.1594548 | 0.313 |
| covid_impact | 1 | 0.0228344 | 0.0081364 | 1.1794643 | 0.304 |
| Adversity:log_cortisol | 1 | 0.0181315 | 0.0064607 | 0.9365480 | 0.427 |
| Residual | 127 | 2.4587122 | 0.8760963 | NA | NA |
| Total | 140 | 2.8064406 | 1.0000000 | NA | NA |

**Supplement 11 – Full model results**

**Supplementary Tables S11.1-11.4**

These tables show the full results of the models testing whether the interaction between CA group and cortisol was associated with alpha diversity.

**Table S11.1 – Faith’s Phylogenetic Diversity**

| **term** | **estimate** | **std_error** | **statistic** | **p_value** | **lower_ci** | **upper_ci** |
| --- | --- | --- | --- | --- | --- | --- |
| intercept | 10.703 | 0.948 | 11.286 | 0.000 | 8.826 | 12.580 |
| child_age_years | 0.077 | 0.049 | 1.569 | 0.119 | -0.020 | 0.174 |
| child_sex: male | 0.313 | 0.331 | 0.945 | 0.347 | -0.342 | 0.968 |
| breastfeeding: combination | 0.160 | 0.487 | 0.329 | 0.742 | -0.804 | 1.125 |
| breastfeeding: DK or NA | 1.004 | 0.813 | 1.235 | 0.219 | -0.605 | 2.614 |
| breastfeeding: formula | 1.036 | 0.618 | 1.676 | 0.096 | -0.187 | 2.258 |
| c_section: No | -0.461 | 0.563 | -0.818 | 0.415 | -1.575 | 0.654 |
| c_section: Yes | -0.128 | 0.597 | -0.215 | 0.830 | -1.310 | 1.053 |
| antibiotics: No | 0.662 | 0.502 | 1.319 | 0.190 | -0.331 | 1.654 |
| antibiotics: Yes | 0.665 | 0.777 | 0.855 | 0.394 | -0.874 | 2.203 |
| covid_impact | 0.031 | 0.043 | 0.715 | 0.476 | -0.055 | 0.117 |
| Adversity: Caregiving Adversity | -1.715 | 0.924 | -1.857 | 0.066 | -3.543 | 0.113 |
| log_cortisol | -0.652 | 0.226 | -2.882 | 0.005 | -1.100 | -0.205 |
| Adversity: Caregiving Adversity:log_cortisol | 0.821 | 0.367 | 2.236 | 0.027 | 0.095 | 1.548 |

**Table S11.2 – Observed Feature Counts**

| **term** | **estimate** | **std_error** | **statistic** | **p_value** | **lower_ci** | **upper_ci** |
| --- | --- | --- | --- | --- | --- | --- |
| intercept | 86.410 | 11.246 | 7.684 | 0.000 | 64.157 | 108.663 |
| child_age_years | 1.445 | 0.581 | 2.487 | 0.014 | 0.295 | 2.596 |
| child_sex: male | 3.111 | 3.926 | 0.792 | 0.430 | -4.657 | 10.879 |
| breastfeeding: combination | -5.601 | 5.778 | -0.969 | 0.334 | -17.034 | 5.832 |
| breastfeeding: DK or NA | 18.900 | 9.644 | 1.960 | 0.052 | -0.183 | 37.983 |
| breastfeeding: formula | 13.711 | 7.326 | 1.871 | 0.064 | -0.786 | 28.209 |
| c_section: No | 3.051 | 6.678 | 0.457 | 0.649 | -10.164 | 16.266 |
| c_section: Yes | 4.815 | 7.079 | 0.680 | 0.498 | -9.194 | 18.824 |
| antibiotics: No | 8.777 | 5.949 | 1.475 | 0.143 | -2.995 | 20.549 |
| antibiotics: Yes | 13.972 | 9.219 | 1.516 | 0.132 | -4.270 | 32.214 |
| covid_impact | 0.415 | 0.515 | 0.807 | 0.421 | -0.603 | 1.434 |
| Adversity: Caregiving Adversity | -17.543 | 10.955 | -1.601 | 0.112 | -39.222 | 4.135 |
| log_cortisol | -6.413 | 2.684 | -2.389 | 0.018 | -11.725 | -1.102 |
| Adversity: Caregiving Adversity:log_cortisol | 7.565 | 4.355 | 1.737 | 0.085 | -1.052 | 16.182 |

**Table S11.3 – Pielou’s Evenness**

| **term** | **estimate** | **std_error** | **statistic** | **p_value** | **lower_ci** | **upper_ci** |
| --- | --- | --- | --- | --- | --- | --- |
| intercept | 0.664 | 0.025 | 26.343 | 0.000 | 0.614 | 0.714 |
| child_age_years | 0.002 | 0.001 | 1.323 | 0.188 | -0.001 | 0.004 |
| child_sex: male | 0.006 | 0.009 | 0.703 | 0.483 | -0.011 | 0.024 |
| breastfeeding: combination | -0.012 | 0.013 | -0.954 | 0.342 | -0.038 | 0.013 |
| breastfeeding: DK or NA | 0.018 | 0.022 | 0.832 | 0.407 | -0.025 | 0.061 |
| breastfeeding: formula | 0.010 | 0.016 | 0.599 | 0.550 | -0.023 | 0.042 |
| c_section: No | 0.006 | 0.015 | 0.368 | 0.714 | -0.024 | 0.035 |
| c_section: Yes | 0.000 | 0.016 | 0.015 | 0.988 | -0.031 | 0.032 |
| antibiotics: No | 0.014 | 0.013 | 1.014 | 0.312 | -0.013 | 0.040 |
| antibiotics: Yes | -0.010 | 0.021 | -0.462 | 0.645 | -0.050 | 0.031 |
| covid_impact | 0.002 | 0.001 | 2.095 | 0.038 | 0.000 | 0.005 |
| Adversity: Caregiving Adversity | 0.034 | 0.025 | 1.398 | 0.165 | -0.014 | 0.083 |
| log_cortisol | 0.009 | 0.006 | 1.543 | 0.125 | -0.003 | 0.021 |
| Adversity: Caregiving Adversity:log_cortisol | -0.011 | 0.010 | -1.161 | 0.248 | -0.031 | 0.008 |

**Table S11.4 – Shannon’s Entropy**

| **term** | **estimate** | **std_error** | **statistic** | **p_value** | **lower_ci** | **upper_ci** |
| --- | --- | --- | --- | --- | --- | --- |
| intercept | 4.248 | 0.235 | 18.051 | 0.000 | 3.782 | 4.713 |
| child_age_years | 0.026 | 0.012 | 2.149 | 0.034 | 0.002 | 0.050 |
| child_sex: male | 0.075 | 0.082 | 0.913 | 0.363 | -0.088 | 0.238 |
| breastfeeding: combination | -0.148 | 0.121 | -1.225 | 0.223 | -0.387 | 0.091 |
| breastfeeding: DK or NA | 0.317 | 0.202 | 1.570 | 0.119 | -0.083 | 0.716 |
| breastfeeding: formula | 0.208 | 0.153 | 1.354 | 0.178 | -0.096 | 0.511 |
| c_section: No | 0.073 | 0.140 | 0.519 | 0.604 | -0.204 | 0.349 |
| c_section: Yes | 0.054 | 0.148 | 0.368 | 0.714 | -0.239 | 0.348 |
| antibiotics: No | 0.172 | 0.124 | 1.384 | 0.169 | -0.074 | 0.419 |
| antibiotics: Yes | 0.072 | 0.193 | 0.373 | 0.710 | -0.310 | 0.454 |
| covid_impact | 0.022 | 0.011 | 1.999 | 0.048 | 0.000 | 0.043 |
| Adversity: Caregiving Adversity | 0.035 | 0.229 | 0.152 | 0.880 | -0.419 | 0.488 |
| log_cortisol | -0.002 | 0.056 | -0.044 | 0.965 | -0.114 | 0.109 |
| Adversity: Caregiving Adversity:log_cortisol | 0.007 | 0.091 | 0.081 | 0.936 | -0.173 | 0.188 |

**Supplementary Tables S11.5-11.8**

These tables show the full results of the models testing whether the interaction between CA group and cortisol was associated with diversity.

**Table S11.5 – Bray-Curtis**

|  | **Df** | **SumOfSqs** | **R2** | **F** | **Pr(>F)** |
| --- | --- | --- | --- | --- | --- |
| child_age_years | 1 | 0.2701363 | 0.0125739 | 1.8061849 | 0.043 |
| child_sex | 1 | 0.1417182 | 0.0065965 | 0.9475562 | 0.464 |
| breastfeeding | 3 | 0.5082191 | 0.0236557 | 1.1326845 | 0.244 |
| c_section | 2 | 0.3631904 | 0.0169052 | 1.2141814 | 0.191 |
| antibiotics | 2 | 0.4174970 | 0.0194329 | 1.3957338 | 0.087 |
| covid_impact | 1 | 0.1647393 | 0.0076680 | 1.1014798 | 0.326 |
| Adversity | 1 | 0.3073635 | 0.0143066 | 2.0550928 | 0.016 |
| log_cortisol | 1 | 0.1361215 | 0.0063360 | 0.9101352 | 0.501 |
| Adversity:log_cortisol | 1 | 0.1806395 | 0.0084081 | 1.2077916 | 0.255 |
| Residual | 127 | 18.9943532 | 0.8841171 | NA | NA |
| Total | 140 | 21.4839781 | 1.0000000 | NA | NA |

**Table S11.6 – Jaccard**

|  | **Df** | **SumOfSqs** | **R2** | **F** | **Pr(>F)** |
| --- | --- | --- | --- | --- | --- |
| child_age_years | 1 | 0.4497731 | 0.0155658 | 2.2351310 | 0.001 |
| child_sex | 1 | 0.1942925 | 0.0067241 | 0.9655293 | 0.546 |
| breastfeeding | 3 | 0.7478732 | 0.0258825 | 1.2388430 | 0.017 |
| c_section | 2 | 0.4831435 | 0.0167207 | 1.2004821 | 0.051 |
| antibiotics | 2 | 0.4405637 | 0.0152471 | 1.0946826 | 0.187 |
| covid_impact | 1 | 0.2156601 | 0.0074636 | 1.0717149 | 0.261 |
| Adversity | 1 | 0.2948671 | 0.0102048 | 1.4653315 | 0.025 |
| log_cortisol | 1 | 0.2449014 | 0.0084756 | 1.2170289 | 0.110 |
| Adversity:log_cortisol | 1 | 0.2677432 | 0.0092661 | 1.3305399 | 0.039 |
| Residual | 127 | 25.5560766 | 0.8844496 | NA | NA |
| Total | 140 | 28.8948943 | 1.0000000 | NA | NA |

**Table S11.7 – Unweighted UniFrac**

|  | **Df** | **SumOfSqs** | **R2** | **F** | **Pr(>F)** |
| --- | --- | --- | --- | --- | --- |
| child_age_years | 1 | 0.1411539 | 0.0169980 | 2.4193384 | 0.003 |
| child_sex | 1 | 0.0511375 | 0.0061581 | 0.8764822 | 0.543 |
| breastfeeding | 3 | 0.1970890 | 0.0237338 | 1.1260172 | 0.242 |
| c_section | 2 | 0.1011387 | 0.0121793 | 0.8667452 | 0.676 |
| antibiotics | 2 | 0.1275388 | 0.0153584 | 1.0929900 | 0.318 |
| covid_impact | 1 | 0.0402378 | 0.0048455 | 0.6896655 | 0.825 |
| Adversity | 1 | 0.0755132 | 0.0090934 | 1.2942762 | 0.183 |
| log_cortisol | 1 | 0.0631259 | 0.0076017 | 1.0819603 | 0.341 |
| Adversity:log_cortisol | 1 | 0.0975332 | 0.0117451 | 1.6716932 | 0.055 |
| Residual | 127 | 7.4096864 | 0.8922867 | NA | NA |
| Total | 140 | 8.3041544 | 1.0000000 | NA | NA |

**Table S11.8 – Weighted UniFrac**

|  | **Df** | **SumOfSqs** | **R2** | **F** | **Pr(>F)** |
| --- | --- | --- | --- | --- | --- |
| child_age_years | 1 | 0.0392532 | 0.0139868 | 2.0275493 | 0.068 |
| child_sex | 1 | 0.0130033 | 0.0046334 | 0.6716588 | 0.628 |
| breastfeeding | 3 | 0.0451719 | 0.0160958 | 0.7777556 | 0.663 |
| c_section | 2 | 0.0716112 | 0.0255167 | 1.8494689 | 0.063 |
| antibiotics | 2 | 0.0486525 | 0.0173360 | 1.2565262 | 0.261 |
| covid_impact | 1 | 0.0299247 | 0.0106629 | 1.5457036 | 0.180 |
| Adversity | 1 | 0.0446214 | 0.0158996 | 2.3048317 | 0.047 |
| log_cortisol | 1 | 0.0373586 | 0.0133117 | 1.9296872 | 0.117 |
| Adversity:log_cortisol | 1 | 0.0181315 | 0.0064607 | 0.9365480 | 0.427 |
| Residual | 127 | 2.4587122 | 0.8760963 | NA | NA |
| Total | 140 | 2.8064406 | 1.0000000 | NA | NA |

**Supplementary Tables S11.9-11.14**

These tables test the associations between each of the 3 health outcomes tested (fatigue, somatic complaints, and internalizing symptoms) and Faith’s Phylogenetic Diversity (11.9-11.11) and observed feature counts (11.12-11.14).

**11.9 – Faith’s Phylogenetic Diversity – Fatigue**

| **term** | **estimate** | **std_error** | **statistic** | **p_value** | **lower_ci** | **upper_ci** |
| --- | --- | --- | --- | --- | --- | --- |
| intercept | 10.502 | 0.971 | 10.818 | 0.000 | 8.581 | 12.424 |
| Adversity: Caregiving Adversity | -1.809 | 0.988 | -1.831 | 0.069 | -3.764 | 0.146 |
| log_cortisol | -0.627 | 0.228 | -2.751 | 0.007 | -1.078 | -0.176 |
| child_age_years | 0.070 | 0.050 | 1.399 | 0.164 | -0.029 | 0.168 |
| child_sex: male | 0.253 | 0.336 | 0.751 | 0.454 | -0.413 | 0.918 |
| breastfeeding: combination | 0.190 | 0.489 | 0.389 | 0.698 | -0.778 | 1.159 |
| breastfeeding: DK or NA | 1.091 | 0.826 | 1.320 | 0.189 | -0.544 | 2.726 |
| breastfeeding: formula | 1.135 | 0.626 | 1.814 | 0.072 | -0.104 | 2.373 |
| c_section: No | -0.554 | 0.570 | -0.972 | 0.333 | -1.683 | 0.574 |
| c_section: Yes | -0.197 | 0.602 | -0.328 | 0.743 | -1.388 | 0.993 |
| antibiotics: No | 0.795 | 0.516 | 1.540 | 0.126 | -0.227 | 1.816 |
| antibiotics: Yes | 0.902 | 0.802 | 1.124 | 0.263 | -0.685 | 2.489 |
| covid_impact | 0.021 | 0.045 | 0.458 | 0.648 | -0.068 | 0.109 |
| pedsql_fatigue | 0.328 | 0.251 | 1.304 | 0.195 | -0.170 | 0.826 |
| Adversity: Caregiving Adversity:log_cortisol | 0.779 | 0.413 | 1.885 | 0.062 | -0.039 | 1.597 |

**11.10 – Faith’s Phylogenetic Diversity – Somatic Complaints**

| **term** | **estimate** | **std_error** | **statistic** | **p_value** | **lower_ci** | **upper_ci** |
| --- | --- | --- | --- | --- | --- | --- |
| intercept | 10.311 | 0.965 | 10.684 | 0.000 | 8.401 | 12.222 |
| Adversity: Caregiving Adversity | -1.876 | 0.941 | -1.995 | 0.048 | -3.738 | -0.015 |
| log_cortisol | -0.667 | 0.225 | -2.970 | 0.004 | -1.112 | -0.223 |
| child_age_years | 0.060 | 0.050 | 1.205 | 0.230 | -0.038 | 0.158 |
| child_sex: male | 0.204 | 0.334 | 0.611 | 0.542 | -0.458 | 0.866 |
| breastfeeding: combination | 0.195 | 0.486 | 0.402 | 0.689 | -0.766 | 1.156 |
| breastfeeding: DK or NA | 1.636 | 0.852 | 1.920 | 0.057 | -0.051 | 3.324 |
| breastfeeding: formula | 1.391 | 0.649 | 2.144 | 0.034 | 0.107 | 2.675 |
| c_section: No | -0.280 | 0.583 | -0.480 | 0.632 | -1.434 | 0.874 |
| c_section: Yes | 0.159 | 0.617 | 0.258 | 0.797 | -1.063 | 1.381 |
| antibiotics: No | 1.011 | 0.522 | 1.938 | 0.055 | -0.022 | 2.044 |
| antibiotics: Yes | 1.132 | 0.798 | 1.418 | 0.159 | -0.448 | 2.712 |
| covid_impact | 0.027 | 0.044 | 0.613 | 0.541 | -0.060 | 0.114 |
| somatic_complaints | 0.071 | 0.071 | 0.992 | 0.323 | -0.070 | 0.211 |
| Adversity: Caregiving Adversity:log_cortisol | 0.856 | 0.365 | 2.347 | 0.021 | 0.134 | 1.578 |

**11.11 – Faith’s Phylogenetic Diversity –Internalizing Symptoms**

| **term** | **estimate** | **std_error** | **statistic** | **p_value** | **lower_ci** | **upper_ci** |
| --- | --- | --- | --- | --- | --- | --- |
| intercept | 10.206 | 0.966 | 10.564 | 0.000 | 8.294 | 12.119 |
| Adversity: Caregiving Adversity | -1.940 | 0.940 | -2.064 | 0.041 | -3.801 | -0.080 |
| log_cortisol | -0.655 | 0.224 | -2.920 | 0.004 | -1.099 | -0.211 |
| child_age_years | 0.062 | 0.049 | 1.259 | 0.210 | -0.036 | 0.160 |
| child_sex: male | 0.182 | 0.335 | 0.544 | 0.587 | -0.480 | 0.845 |
| breastfeeding: combination | 0.248 | 0.485 | 0.510 | 0.611 | -0.713 | 1.209 |
| breastfeeding: DK or NA | 1.671 | 0.851 | 1.964 | 0.052 | -0.014 | 3.356 |
| breastfeeding: formula | 1.460 | 0.651 | 2.244 | 0.027 | 0.172 | 2.749 |
| c_section: No | -0.283 | 0.581 | -0.488 | 0.626 | -1.433 | 0.866 |
| c_section: Yes | 0.123 | 0.617 | 0.200 | 0.842 | -1.098 | 1.345 |
| antibiotics: No | 1.024 | 0.518 | 1.976 | 0.050 | -0.002 | 2.051 |
| antibiotics: Yes | 1.250 | 0.804 | 1.555 | 0.122 | -0.341 | 2.841 |
| covid_impact | 0.021 | 0.045 | 0.460 | 0.646 | -0.068 | 0.110 |
| internalizing | 0.032 | 0.022 | 1.458 | 0.147 | -0.012 | 0.076 |
| Adversity: Caregiving Adversity:log_cortisol | 0.846 | 0.364 | 2.321 | 0.022 | 0.124 | 1.567 |

**11.12 – Observed Feature Counts – Fatigue**

| **term** | **estimate** | **std_error** | **statistic** | **p_value** | **lower_ci** | **upper_ci** |
| --- | --- | --- | --- | --- | --- | --- |
| intercept | 86.042 | 11.595 | 7.421 | 0.000 | 63.092 | 108.992 |
| Adversity: Caregiving Adversity | -17.333 | 11.797 | -1.469 | 0.144 | -40.682 | 6.017 |
| log_cortisol | -6.335 | 2.723 | -2.327 | 0.022 | -11.724 | -0.946 |
| child_age_years | 1.411 | 0.595 | 2.373 | 0.019 | 0.234 | 2.588 |
| child_sex: male | 2.913 | 4.017 | 0.725 | 0.470 | -5.037 | 10.863 |
| breastfeeding: combination | -5.498 | 5.846 | -0.940 | 0.349 | -17.069 | 6.073 |
| breastfeeding: DK or NA | 19.200 | 9.866 | 1.946 | 0.054 | -0.328 | 38.728 |
| breastfeeding: formula | 13.971 | 7.472 | 1.870 | 0.064 | -0.819 | 28.760 |
| c_section: No | 2.781 | 6.812 | 0.408 | 0.684 | -10.701 | 16.263 |
| c_section: Yes | 4.609 | 7.186 | 0.641 | 0.522 | -9.614 | 18.832 |
| antibiotics: No | 9.101 | 6.162 | 1.477 | 0.142 | -3.097 | 21.298 |
| antibiotics: Yes | 14.624 | 9.576 | 1.527 | 0.129 | -4.329 | 33.577 |
| covid_impact | 0.381 | 0.536 | 0.711 | 0.478 | -0.679 | 1.441 |
| pedsql_fatigue | 0.952 | 3.003 | 0.317 | 0.752 | -4.992 | 6.895 |
| Adversity: Caregiving Adversity:log_cortisol | 7.180 | 4.937 | 1.454 | 0.148 | -2.591 | 16.951 |

**11.13 – Observed Feature Counts – Somatic Complaints**

| **term** | **estimate** | **std_error** | **statistic** | **p_value** | **lower_ci** | **upper_ci** |
| --- | --- | --- | --- | --- | --- | --- |
| intercept | 82.259 | 11.559 | 7.116 | 0.000 | 59.378 | 105.139 |
| Adversity: Caregiving Adversity | -18.060 | 11.265 | -1.603 | 0.111 | -40.359 | 4.238 |
| log_cortisol | -6.541 | 2.691 | -2.431 | 0.017 | -11.867 | -1.215 |
| child_age_years | 1.317 | 0.593 | 2.220 | 0.028 | 0.143 | 2.490 |
| child_sex: male | 2.227 | 4.006 | 0.556 | 0.579 | -5.702 | 10.157 |
| breastfeeding: combination | -5.012 | 5.816 | -0.862 | 0.391 | -16.523 | 6.500 |
| breastfeeding: DK or NA | 24.554 | 10.210 | 2.405 | 0.018 | 4.345 | 44.764 |
| breastfeeding: formula | 16.863 | 7.770 | 2.170 | 0.032 | 1.483 | 32.244 |
| c_section: No | 5.563 | 6.983 | 0.797 | 0.427 | -8.259 | 19.384 |
| c_section: Yes | 8.108 | 7.391 | 1.097 | 0.275 | -6.523 | 22.739 |
| antibiotics: No | 11.511 | 6.249 | 1.842 | 0.068 | -0.857 | 23.880 |
| antibiotics: Yes | 17.632 | 9.560 | 1.844 | 0.068 | -1.291 | 36.555 |
| covid_impact | 0.413 | 0.527 | 0.783 | 0.435 | -0.630 | 1.456 |
| somatic_complaints | 0.228 | 0.851 | 0.268 | 0.789 | -1.457 | 1.914 |
| Adversity: Caregiving Adversity:log_cortisol | 7.909 | 4.369 | 1.810 | 0.073 | -0.739 | 16.558 |

**11.14 – Observed Feature Counts – Internalizing Symptoms**

| **term** | **estimate** | **std_error** | **statistic** | **p_value** | **lower_ci** | **upper_ci** |
| --- | --- | --- | --- | --- | --- | --- |
| intercept | 81.419 | 11.581 | 7.030 | 0.000 | 58.493 | 104.345 |
| Adversity: Caregiving Adversity | -19.387 | 11.266 | -1.721 | 0.088 | -41.690 | 2.916 |
| log_cortisol | -6.456 | 2.690 | -2.400 | 0.018 | -11.781 | -1.131 |
| child_age_years | 1.310 | 0.593 | 2.211 | 0.029 | 0.137 | 2.484 |
| child_sex: male | 1.881 | 4.012 | 0.469 | 0.640 | -6.062 | 9.823 |
| breastfeeding: combination | -4.770 | 5.819 | -0.820 | 0.414 | -16.291 | 6.750 |
| breastfeeding: DK or NA | 25.261 | 10.201 | 2.476 | 0.015 | 5.067 | 45.455 |
| breastfeeding: formula | 17.710 | 7.800 | 2.271 | 0.025 | 2.269 | 33.151 |
| c_section: No | 5.002 | 6.959 | 0.719 | 0.474 | -8.774 | 18.779 |
| c_section: Yes | 7.522 | 7.394 | 1.017 | 0.311 | -7.115 | 22.158 |
| antibiotics: No | 12.237 | 6.215 | 1.969 | 0.051 | -0.066 | 24.540 |
| antibiotics: Yes | 19.469 | 9.634 | 2.021 | 0.045 | 0.398 | 38.540 |
| covid_impact | 0.328 | 0.539 | 0.608 | 0.544 | -0.739 | 1.395 |
| internalizing | 0.281 | 0.266 | 1.057 | 0.293 | -0.245 | 0.808 |
| Adversity: Caregiving Adversity:log_cortisol | 7.804 | 4.368 | 1.787 | 0.076 | -0.843 | 16.451 |

**Supplementary Tables S11.15-11.26**

These tables test the associations between each of the 3 health outcomes tested (fatigue, somatic complaints, and internalizing symptoms) and Bray-Curtis dissimilarity (11.15-11.17), Jaccard dissimilarity (11.18-11.20), Unweighted UniFrac dissimilarity (11.21-11.23), and Weighted UniFrac dissimilarity (11.24-11.26).

**Table S11.15 – Bray-Curtis – Fatigue**

|  | **Df** | **SumOfSqs** | **R2** | **F** | **Pr(>F)** |
| --- | --- | --- | --- | --- | --- |
| child_age_years | 1 | 0.2594084 | 0.0123165 | 1.7425712 | 0.052 |
| child_sex | 1 | 0.1418900 | 0.0067368 | 0.9531433 | 0.451 |
| breastfeeding | 3 | 0.4918824 | 0.0233542 | 1.1014036 | 0.291 |
| c_section | 2 | 0.3662398 | 0.0173888 | 1.2301044 | 0.191 |
| antibiotics | 2 | 0.4177739 | 0.0198356 | 1.4031943 | 0.088 |
| covid_impact | 1 | 0.1773135 | 0.0084187 | 1.1911005 | 0.231 |
| Adversity | 1 | 0.3074870 | 0.0145993 | 2.0655385 | 0.019 |
| log_cortisol | 1 | 0.1169026 | 0.0055505 | 0.7852913 | 0.687 |
| pedsql_fatigue | 1 | 0.1438278 | 0.0068288 | 0.9661607 | 0.441 |
| Adversity:log_cortisol | 1 | 0.1797957 | 0.0085366 | 1.2077740 | 0.217 |
| Residual | 124 | 18.4592986 | 0.8764342 | NA | NA |
| Total | 138 | 21.0618197 | 1.0000000 | NA | NA |

**Table S11.16 – Bray-Curtis – Somatic Complaints**

|  | **Df** | **SumOfSqs** | **R2** | **F** | **Pr(>F)** |
| --- | --- | --- | --- | --- | --- |
| child_age_years | 1 | 0.2660533 | 0.0126150 | 1.7664923 | 0.039 |
| child_sex | 1 | 0.1566579 | 0.0074280 | 1.0401482 | 0.396 |
| breastfeeding | 3 | 0.4831439 | 0.0229083 | 1.0692969 | 0.333 |
| c_section | 2 | 0.3944338 | 0.0187021 | 1.3094445 | 0.144 |
| antibiotics | 2 | 0.3889301 | 0.0184412 | 1.2911736 | 0.135 |
| covid_impact | 1 | 0.1939270 | 0.0091951 | 1.2876009 | 0.171 |
| Adversity | 1 | 0.2757901 | 0.0130766 | 1.8311404 | 0.038 |
| log_cortisol | 1 | 0.1302372 | 0.0061752 | 0.8647248 | 0.574 |
| somatic_complaints | 1 | 0.0942489 | 0.0044688 | 0.6257768 | 0.891 |
| Adversity:log_cortisol | 1 | 0.1817176 | 0.0086162 | 1.2065350 | 0.206 |
| Residual | 123 | 18.5251653 | 0.8783735 | NA | NA |
| Total | 137 | 21.0903051 | 1.0000000 | NA | NA |

**Table S11.17 – Bray-Curtis – Internalizing Symptoms**

|  | **Df** | **SumOfSqs** | **R2** | **F** | **Pr(>F)** |
| --- | --- | --- | --- | --- | --- |
| child_age_years | 1 | 0.2642217 | 0.0125938 | 1.7466406 | 0.049 |
| child_sex | 1 | 0.1545290 | 0.0073655 | 1.0215157 | 0.405 |
| breastfeeding | 3 | 0.4789746 | 0.0228298 | 1.0554222 | 0.346 |
| c_section | 2 | 0.3853109 | 0.0183654 | 1.2735512 | 0.137 |
| antibiotics | 2 | 0.3902219 | 0.0185995 | 1.2897830 | 0.123 |
| covid_impact | 1 | 0.2001559 | 0.0095402 | 1.3231329 | 0.164 |
| Adversity | 1 | 0.2762009 | 0.0131648 | 1.8258289 | 0.040 |
| log_cortisol | 1 | 0.1302316 | 0.0062073 | 0.8608976 | 0.570 |
| internalizing | 1 | 0.0627985 | 0.0029932 | 0.4151301 | 0.988 |
| Adversity:log_cortisol | 1 | 0.1821372 | 0.0086814 | 1.2040200 | 0.240 |
| Residual | 122 | 18.4554560 | 0.8796590 | NA | NA |
| Total | 136 | 20.9802382 | 1.0000000 | NA | NA |

**Table S11.18 – Jaccard – Fatigue**

|  | **Df** | **SumOfSqs** | **R2** | **F** | **Pr(>F)** |
| --- | --- | --- | --- | --- | --- |
| child_age_years | 1 | 0.4463729 | 0.0157041 | 2.2181086 | 0.001 |
| child_sex | 1 | 0.1962374 | 0.0069039 | 0.9751394 | 0.501 |
| breastfeeding | 3 | 0.7487837 | 0.0263434 | 1.2402811 | 0.024 |
| c_section | 2 | 0.4824192 | 0.0169723 | 1.1986147 | 0.071 |
| antibiotics | 2 | 0.4393954 | 0.0154586 | 1.0917180 | 0.221 |
| covid_impact | 1 | 0.2160523 | 0.0076011 | 1.0736036 | 0.289 |
| Adversity | 1 | 0.2948245 | 0.0103724 | 1.4650370 | 0.017 |
| log_cortisol | 1 | 0.2449875 | 0.0086190 | 1.2173878 | 0.129 |
| pedsql_fatigue | 1 | 0.1703291 | 0.0059924 | 0.8463965 | 0.830 |
| Adversity:log_cortisol | 1 | 0.2307633 | 0.0081186 | 1.1467049 | 0.171 |
| Residual | 124 | 24.9538003 | 0.8779141 | NA | NA |
| Total | 138 | 28.4239657 | 1.0000000 | NA | NA |

**Table S11.19 – Jaccard – Somatic Complaints**

|  | **Df** | **SumOfSqs** | **R2** | **F** | **Pr(>F)** |
| --- | --- | --- | --- | --- | --- |
| child_age_years | 1 | 0.4382402 | 0.0154771 | 2.180370 | 0.001 |
| child_sex | 1 | 0.2049349 | 0.0072376 | 1.019610 | 0.379 |
| breastfeeding | 3 | 0.7394096 | 0.0261133 | 1.226258 | 0.019 |
| c_section | 2 | 0.4937550 | 0.0174377 | 1.228286 | 0.052 |
| antibiotics | 2 | 0.4516724 | 0.0159514 | 1.123600 | 0.147 |
| covid_impact | 1 | 0.2196289 | 0.0077565 | 1.092716 | 0.260 |
| Adversity | 1 | 0.2918716 | 0.0103079 | 1.452145 | 0.017 |
| log_cortisol | 1 | 0.2417058 | 0.0085362 | 1.202556 | 0.123 |
| somatic_complaints | 1 | 0.2408644 | 0.0085065 | 1.198369 | 0.132 |
| Adversity:log_cortisol | 1 | 0.2711611 | 0.0095764 | 1.349104 | 0.044 |
| Residual | 123 | 24.7222024 | 0.8730995 | NA | NA |
| Total | 137 | 28.3154465 | 1.0000000 | NA | NA |

**Table S11.20 – Jaccard – Internalizing Symptoms**

|  | **Df** | **SumOfSqs** | **R2** | **F** | **Pr(>F)** |
| --- | --- | --- | --- | --- | --- |
| child_age_years | 1 | 0.4315067 | 0.0153513 | 2.1425202 | 0.001 |
| child_sex | 1 | 0.2027702 | 0.0072138 | 1.0067961 | 0.453 |
| breastfeeding | 3 | 0.7408023 | 0.0263548 | 1.2260789 | 0.023 |
| c_section | 2 | 0.4911453 | 0.0174730 | 1.2193191 | 0.041 |
| antibiotics | 2 | 0.4514683 | 0.0160615 | 1.1208168 | 0.171 |
| covid_impact | 1 | 0.2225586 | 0.0079178 | 1.1050495 | 0.230 |
| Adversity | 1 | 0.2923306 | 0.0104000 | 1.4514820 | 0.018 |
| log_cortisol | 1 | 0.2416911 | 0.0085984 | 1.2000462 | 0.137 |
| internalizing | 1 | 0.1936677 | 0.0068899 | 0.9616002 | 0.526 |
| Adversity:log_cortisol | 1 | 0.2698788 | 0.0096012 | 1.3400042 | 0.035 |
| Residual | 122 | 24.5709781 | 0.8741384 | NA | NA |
| Total | 136 | 28.1087976 | 1.0000000 | NA | NA |

**Table S11.21 – Unweighted UniFrac – Fatigue**

|  | **Df** | **SumOfSqs** | **R2** | **F** | **Pr(>F)** |
| --- | --- | --- | --- | --- | --- |
| child_age_years | 1 | 0.1373299 | 0.0167531 | 2.3437008 | 0.007 |
| child_sex | 1 | 0.0514275 | 0.0062737 | 0.8776733 | 0.564 |
| breastfeeding | 3 | 0.2014180 | 0.0245713 | 1.1458143 | 0.225 |
| c_section | 2 | 0.1019023 | 0.0124312 | 0.8695431 | 0.630 |
| antibiotics | 2 | 0.1265301 | 0.0154356 | 1.0796946 | 0.325 |
| covid_impact | 1 | 0.0391081 | 0.0047709 | 0.6674273 | 0.834 |
| Adversity | 1 | 0.0755423 | 0.0092155 | 1.2892208 | 0.198 |
| log_cortisol | 1 | 0.0576273 | 0.0070301 | 0.9834792 | 0.424 |
| pedsql_fatigue | 1 | 0.0586776 | 0.0071582 | 1.0014038 | 0.419 |
| Adversity:log_cortisol | 1 | 0.0818917 | 0.0099901 | 1.3975806 | 0.122 |
| Residual | 124 | 7.2658179 | 0.8863702 | NA | NA |
| Total | 138 | 8.1972726 | 1.0000000 | NA | NA |

**Table S11.22 – Unweighted UniFrac – Somatic Complaints**

|  | **Df** | **SumOfSqs** | **R2** | **F** | **Pr(>F)** |
| --- | --- | --- | --- | --- | --- |
| child_age_years | 1 | 0.1314752 | 0.0162023 | 2.2656863 | 0.010 |
| child_sex | 1 | 0.0506703 | 0.0062443 | 0.8731908 | 0.583 |
| breastfeeding | 3 | 0.2025353 | 0.0249594 | 1.1634169 | 0.209 |
| c_section | 2 | 0.1049868 | 0.0129380 | 0.9046082 | 0.600 |
| antibiotics | 2 | 0.1370973 | 0.0168952 | 1.1812854 | 0.204 |
| covid_impact | 1 | 0.0401068 | 0.0049426 | 0.6911519 | 0.816 |
| Adversity | 1 | 0.0864157 | 0.0106494 | 1.4891845 | 0.099 |
| log_cortisol | 1 | 0.0610445 | 0.0075228 | 1.0519679 | 0.363 |
| somatic_complaints | 1 | 0.0615949 | 0.0075906 | 1.0614525 | 0.348 |
| Adversity:log_cortisol | 1 | 0.1011104 | 0.0124603 | 1.7424149 | 0.049 |
| Residual | 123 | 7.1375502 | 0.8795950 | NA | NA |
| Total | 137 | 8.1145873 | 1.0000000 | NA | NA |

**Table S11.23 – Unweighted UniFrac – Internalizing Symptoms**

|  | **Df** | **SumOfSqs** | **R2** | **F** | **Pr(>F)** |
| --- | --- | --- | --- | --- | --- |
| child_age_years | 1 | 0.1292803 | 0.0160459 | 2.2230446 | 0.013 |
| child_sex | 1 | 0.0494776 | 0.0061410 | 0.8507937 | 0.622 |
| breastfeeding | 3 | 0.1997425 | 0.0247915 | 1.1448932 | 0.211 |
| c_section | 2 | 0.1032779 | 0.0128186 | 0.8879596 | 0.628 |
| antibiotics | 2 | 0.1370507 | 0.0170103 | 1.1783300 | 0.228 |
| covid_impact | 1 | 0.0402848 | 0.0050000 | 0.6927195 | 0.815 |
| Adversity | 1 | 0.0864107 | 0.0107251 | 1.4858792 | 0.093 |
| log_cortisol | 1 | 0.0610464 | 0.0075769 | 1.0497262 | 0.350 |
| internalizing | 1 | 0.0552893 | 0.0068624 | 0.9507300 | 0.479 |
| Adversity:log_cortisol | 1 | 0.1001756 | 0.0124335 | 1.7225726 | 0.044 |
| Residual | 122 | 7.0948635 | 0.8805948 | NA | NA |
| Total | 136 | 8.0568994 | 1.0000000 | NA | NA |

**Table S11.24 – Weighted UniFrac – Fatigue**

|  | **Df** | **SumOfSqs** | **R2** | **F** | **Pr(>F)** |
| --- | --- | --- | --- | --- | --- |
| child_age_years | 1 | 0.0360927 | 0.0131498 | 1.8691138 | 0.125 |
| child_sex | 1 | 0.0143311 | 0.0052213 | 0.7421590 | 0.556 |
| breastfeeding | 3 | 0.0406223 | 0.0148001 | 0.7012292 | 0.724 |
| c_section | 2 | 0.0736715 | 0.0268410 | 1.9075953 | 0.059 |
| antibiotics | 2 | 0.0489998 | 0.0178523 | 1.2687651 | 0.239 |
| covid_impact | 1 | 0.0390633 | 0.0142321 | 2.0229512 | 0.083 |
| Adversity | 1 | 0.0444973 | 0.0162119 | 2.3043592 | 0.064 |
| log_cortisol | 1 | 0.0178589 | 0.0065066 | 0.9248509 | 0.406 |
| pedsql_fatigue | 1 | 0.0132720 | 0.0048355 | 0.6873128 | 0.574 |
| Adversity:log_cortisol | 1 | 0.0218803 | 0.0079718 | 1.1331069 | 0.283 |
| Residual | 124 | 2.3944446 | 0.8723777 | NA | NA |
| Total | 138 | 2.7447337 | 1.0000000 | NA | NA |

**Table S11.25 – Weighted UniFrac – Somatic Complaints**

|  | **Df** | **SumOfSqs** | **R2** | **F** | **Pr(>F)** |
| --- | --- | --- | --- | --- | --- |
| child_age_years | 1 | 0.0337390 | 0.0122661 | 1.7343705 | 0.117 |
| child_sex | 1 | 0.0154714 | 0.0056248 | 0.7953163 | 0.536 |
| breastfeeding | 3 | 0.0389525 | 0.0141616 | 0.6674577 | 0.798 |
| c_section | 2 | 0.0833146 | 0.0302898 | 2.1414161 | 0.032 |
| antibiotics | 2 | 0.0464171 | 0.0168754 | 1.1930491 | 0.297 |
| covid_impact | 1 | 0.0380658 | 0.0138392 | 1.9567925 | 0.109 |
| Adversity | 1 | 0.0404202 | 0.0146951 | 2.0778202 | 0.090 |
| log_cortisol | 1 | 0.0343041 | 0.0124716 | 1.7634229 | 0.126 |
| somatic_complaints | 1 | 0.0088242 | 0.0032081 | 0.4536133 | 0.788 |
| Adversity:log_cortisol | 1 | 0.0183315 | 0.0066646 | 0.9423413 | 0.396 |
| Residual | 123 | 2.3927377 | 0.8699036 | NA | NA |
| Total | 137 | 2.7505780 | 1.0000000 | NA | NA |

**Table S11.26 – Weighted UniFrac – Somatic Complaints**

|  | **Df** | **SumOfSqs** | **R2** | **F** | **Pr(>F)** |
| --- | --- | --- | --- | --- | --- |
| child_age_years | 1 | 0.0349554 | 0.0127500 | 1.7846921 | 0.113 |
| child_sex | 1 | 0.0150889 | 0.0055037 | 0.7703851 | 0.532 |
| breastfeeding | 3 | 0.0380957 | 0.0138955 | 0.6483411 | 0.793 |
| c_section | 2 | 0.0807767 | 0.0294634 | 2.0620776 | 0.036 |
| antibiotics | 2 | 0.0459878 | 0.0167741 | 1.1739815 | 0.290 |
| covid_impact | 1 | 0.0371773 | 0.0135605 | 1.8981351 | 0.112 |
| Adversity | 1 | 0.0404693 | 0.0147612 | 2.0662089 | 0.071 |
| log_cortisol | 1 | 0.0343003 | 0.0125111 | 1.7512421 | 0.130 |
| internalizing | 1 | 0.0069139 | 0.0025219 | 0.3529994 | 0.881 |
| Adversity:log_cortisol | 1 | 0.0183043 | 0.0066765 | 0.9345513 | 0.408 |
| Residual | 122 | 2.3895216 | 0.8715820 | NA | NA |
| Total | 136 | 2.7415913 | 1.0000000 | NA | NA |
